# High glucose promotes PRDM16 proteolysis and adipose tissue fibrosis

**DOI:** 10.21203/rs.3.rs-11471586/v1

**Published:** 2026-10-30

**Authors:** Yong Chen, Li Chen, Yusuke Higuchi, Daisuke Katoh, Qiang Wang, Shingo Kajimura

**Affiliations:** 1 Division of Endocrinology, Diabetes and Metabolism, Beth Israel Deaconess Medical Center and Harvard Medical School, and Howard Hughes Medical Institute, Boston, MA, USA.; 2 Department of Pathology, Beth Israel Deaconess Medical Center, Harvard Medical School, Boston, MA, USA.; 3 Tsinghua-Peking Center for Life Sciences, School of Life Sciences, Tsinghua University, Beijing, 100084, China.

**Keywords:** Adipose tissue, O-GlcNAcylation, PRDM16, Fibrosis, Glucose homeostasis, UCP1-independent

## Abstract

Chronic nutrient excess drives adipose tissue fibrosis and inflammation, key features of metabolic dysfunction that underlie insulin resistance and Type 2 diabetes. However, the molecular mechanisms linking hyperglycemia to pathological adipose tissue remodeling remain less understood. Here, we identify a post-translational mechanism by which high glucose destabilizes PRDM16, a key transcriptional regulator of adipose tissue identity, through O-GlcNAcylation of its E3 ligase adaptor protein, amyloid beta precursor protein binding protein 2 (APPBP2). Glucose-induced O-GlcNAcylation of APPBP2 at S561 promotes its interaction with PRDM16, triggering PRDM16 ubiquitination and proteasomal degradation. Notably, APPBP2^S561N^ is a hypomorphic variant identified in human populations who exhibit improved glucose homeostasis. Knock-in mice harboring APPBP2^S561N^ maintained elevated PRDM16 protein levels independently of mRNA abundance and are protected against diet-induced adipose tissue fibrosis, inflammation, glucose intolerance, and insulin resistance. These metabolic benefits are independent of UCP1-mediated thermogenesis, as metabolic protection of APPBP2^S561N^ persists in the *Ucp1* null background. These findings reveal a glucose-responsive O-GlcNAc pathway that couples nutrient excess to adipose tissue dysfunction through post-translational regulation of PRDM16 protein stability.

## INTRODUCTION

Chronic hyperglycemia is a hallmark of diabetes and a major driver of tissue damage in multiple organs, including the vasculature, adipose tissue, kidney, retina, and peripheral nerves ^1-4^. In obesity and Type 2 diabetes, adipose tissue undergoes pathological remodeling characterized by adipocyte hypertrophy, chronic low-grade inflammation, excessive deposition of extracellular matrix, and impaired insulin sensitivity ^4-6^. Although adipose tissue dysfunction is often viewed as a consequence of lipid overload, high glucose can itself alter adipocyte function by inducing endoplasmic reticulum (ER) stress, oxidative stress, and pro-inflammatory responses ^7-9^. How chronically elevated glucose is sensed and triggers adipose tissue dysfunction remains a significant area of research.

One proposed mechanism by which cells couple glucose availability to protein function is O-GlcNAcylation, the reversible attachment of a single O-linked β-N-acetylglucosamine (O-GlcNAc) moiety to serine and threonine residues of nucleocytoplasmic proteins ^10-12^. In contrast to the complex glycosylation in the secretory pathway, O-GlcNAcylation is added or removed by a pair of enzymes, O-GlcNAc transferase (OGT) and O-GlcNAcase (OGA), respectively ^13-16^. The hexosamine biosynthetic pathway (HBP), which generates the donor substrate (UDP-GlcNAc) for OGT, is activated in response to elevated glucose levels ^10, 17-19^. In agreement, increased flux through the HBP has been linked to glucose-induced insulin resistance, and elevating O-GlcNAcylation attenuates insulin signaling in adipocytes ^20-23^. *In vivo* mouse models, OGT in the CNS or peripheral tissues regulates insulin resistance, hyperphagia, and obesity ^24-26^. Fat-specific deletion of OGT protects against diet-induced weight gain, in part by blunting diet-induced hyperphagia and enhancing visceral fat lipolysis, yet it also decreases *de novo* lipogenesis and increases inflammation and fibrosis. Furthermore, O-GlcNAcylation has been implicated in regulating protein turnover via the ubiquitin-proteasome system ^30-32^. However, OGT modifies a large number of intracellular proteins, and thus, it remains elusive which O-GlcNAcylated substrates mediate the effects of elevated glucose on adipose tissue dysfunction.

PRDM16 (PR-domain containing protein 16) is a dominant transcriptional regulator of the brown and beige adipocyte gene program by forming a complex with transcriptional and epigenetic regulators ^33-35^. PRDM16 regulates beige adipocyte biogenesis in subcutaneous white adipose tissue (WAT) and the loss of its complex leads to adipose tissue fibrosis and inflammation ^36-39^. Although PRDM16 transcription itself is not dynamically regulated by external or hormonal cues, recent studies showed that its protein stability is dynamically regulated post-translationally. For example, synthetic ligands for peroxisome proliferator-activated receptor-γ (PPARγ), such as thiazolidinediones (TZDs), extend PRDM16 half-life by suppressing its ubiquitin-proteasome degradation ^40^, whereas aging, obesity, and insulin resistance reduce PRDM16 protein stability in adipose tissue ^37, 41, 42^. Mechanistically, our previous study identified an E3 ubiquitin ligase complex of amyloid beta precursor protein binding protein 2 (APPBP2) and Cullin2 (CUL2) that mediates PRDM16 ubiquitination and proteasomal degradation ^41^. Cullin-RING E3 ligases (CRLs) recognize substrates through multifaceted mechanisms, including CRL complex assembly and post-translational modification of the substrate or the ligase itself ^43-45^. However, it remains unknown how the CUL2-APPBP2 complex targets PRDM16 for degradation under pathological conditions, including hyperglycemia.

A clue emerged from experiments on cultured cells incubated under a range of glucose concentrations. We found that PRDM16 protein levels were significantly lower in HEK293 cells ectopically expressing PRDM16 when incubated in high-glucose medium (25 mM) than in low-glucose medium (5.5 mM) (Fig. 1a). This difference was independent of transcription, as glucose concentrations did not affect *Prdm16* mRNA levels (Fig. 1b). These results suggest that a glucose-sensitive, post-translational mechanism regulates PRDM16 protein expression. Thus, the present study elucidated the underlying mechanism linking glucose sensing to PRDM16 protein regulation and adipose tissue remodeling.

## RESULTS

### High glucose promotes PRDM16 protein degradation through the PRDM16-APPBP2 interaction.

Given the role of APPBP2 as a substrate receptor for PRDM16 ^41^, we first examined the extent to which the PRDM16-APPBP2 interaction is regulated by glucose levels. We found that cells incubated in a high-glucose medium (25 mM) for 18 hours showed substantially enhanced interaction between PRDM16 and APPBP2, whereas the interaction was reduced under a low-glucose medium (5.5 mM) (Fig. 1c). On the other hand, we did not find a notable change in endogenous APPBP2 protein expression. We also found that the high-glucose-induced PRDM16-APPBP2 interaction was accompanied by elevated O-GlcNAcylation of intracellular proteins (see input for RL2 immunoblotting, bottom panel in Fig. 1c). Notably, the PRDM16-APPBP2 interaction was enhanced even under a low glucose condition (5.5 mM) when O-GlcNAcase (OGA) was pharmacologically inhibited by Thiamet-G to elevate O-GlcNAcylation in the presence of the proteasome inhibitor MG132 (Fig. 1d; Extended Data Fig.1a).

Thus, we next examined whether glucose regulates PRDM16 protein expression *in vivo*, in which wild-type mice were on a high sucrose water (20%) or no sucrose water (0%, control) for 6 days (Fig. 1e). From Day 3 for an additional 3 days, these mice were treated with a pharmacological OGA inhibitor, Thiamet-G (20 mg/kg) or vehicle (saline), a dose used in a previous study ^47^. At the end of the treatments, we found that PRDM16 protein expression in the inguinal WAT was markedly reduced in mice on a high-sucrose water compared to those on a control water (Fig. 1f, quantification in the right graph). Importantly, treatment with the OGA inhibitor significantly reduced PRDM16 protein expression in the inguinal WAT even without sucrose water treatment (Fig. 1f). By contrast, we found no difference in *Prdm16* mRNA levels in these samples (Extended Data Fig.1b). These results suggested that high-glucose-linked O-GlcNAcylation is involved in the post-transcriptional regulation of PRDM16 protein expression.

To further investigate the underlying mechanisms, we hypothesized that APPBP2 itself undergoes O-GlcNAcylation. This is based on two independent prediction algorithms (O-GlcNAcPRED-DL and DeepO-GlcNAc), indicating that several serine or threonine residues within the APPBP2 are potential O-GlcNAcylation sites (Fig. 1g). Of note, intrinsically disordered region (IDR) analysis revealed that the C-terminal region of APPBP2 (amino acids 556–585) displays strong disorder propensity (Extended Data Fig.1c), a feature frequently associated with post-translational modifications, including O-GlcNAcylation ^48, 49^. Accordingly, we conducted three complementary experiments to test whether APPBP2 undergoes O-GlcNAcylation by OGT. First, we performed *in vitro* O-GlcNAcylation assays using purified APPBP2 and OGT proteins. In the presence of UDP-GlcNAc, Ca^2+^, and Mg^2+^, we found that APPBP2 was O-GlcNAcylated by OGT (Fig. 1h). Second, we immunopurified O-GlcNAcylated proteins from cells expressing HA-tagged APPBP2 and Flag-tagged OGT, following the method to pulldown the wheat germ agglutinin (WGA) ^50^. We found that the HA-APPBP2 protein was highly detected from GlcNAcylated protein precipitants from cells expressing OGT (Fig. 1i). Third, we coexpressed HA-tagged APPBP2 with wild-type OGT (OGT-WT) or the catalytically inactive mutant OGT-K842M. Ectopic expression of the wild-type OGT increased O-GlcNAcylation of APPBP2, as detected by RL2 immunoblotting in immunoprecipitated APPBP2 protein; however, expression of the catalytically inactive mutant OGT-K842M did not elevate APPBP2 O-GlcNAcylation, comparable to the signal from endogenous O-GlcNAcylated APPBP2 (Fig. 1j). Together, these results support O-GlcNAcylation of the APPBP2 protein by OGT.

### Identification of glucose-sensitive O-glycosylation of APPBP2

The above results prompted us to identify the O-GlcNAcylation sites within APPBP2. When we overlapped the *in silico* prediction (shown in Figure 1g) with the IDR prediction (shown in Extended Data Fig.1c), seven candidate residues were identified, including S561, T562, S563, S566, T567, S577, and S584. To functionally interrogate these sites, we generated an APPBP2 mutant in which all these residues were substituted with alanine (ST7A). We found that the ST7A mutant completely abolished the O-GlcNAcylation signal in immunopurified APPBP2, as indicated by the RL2 immunoblotting signal, even when OGT was ectopically expressed (Fig. 2a). Subsequently, we mutated each site of these seven residues to alanine (T to A). We found that Ala mutants at S561, T562, and S563 markedly reduced APPBP2 O-GlcNAcylation, whereas mutations at S566, T567, S577, or S584 had minimal impact (Fig. 2b). Furthermore, the combinatory mutations at S561/T562/S563 to Ala (ST3A) nearly completely eliminated APPBP2 O-GlcNAcylation (Fig. 2c). The data suggest that the clustered Serine/Threonine motif constitutes the major glucose-responsive O-GlcNAcylation region within APPBP2.

The results caught our attention because there is a coding single-nucleotide polymorphism (SNP) in human *APPBP2* (rs34146848) that substitutes serine at 561 with asparagine (S561N). APPBP2 at S561 is evolutionarily conserved across vertebrate species (Fig. 2d). Notably, the Type 2 Diabetes Knowledge Portal suggests that this variant was associated with favorable glucose profiles ^41, 51^; we will discuss this further (Figure 4). These observations raised the possibility that APPBP2 S561 O-GlcNAcylation is functionally coupled to glucose-induced PRDM16 proteolysis. To test this, we ectopically expressed either the wild-type form of APPBP2 (APPBP2-WT) or the APPBP2-S561N mutant in HEK293 cells in a high-glucose medium (25 mM) and examined their O-GlcNAcylation status. We found that the APPBP2-S561N mutant displayed significantly reduced O-GlcNAcylation compared to the wild-type form (Fig. 2e). These data indicate that S561 is a primary, if not the sole, site of O-GlcNAcylation for APPBP2.

We next investigated whether S561 O-GlcNAcylation regulates APPBP2 interaction with PRDM16. Consistent with the above result (see Fig. 1d), overexpression of OGT enhanced the APPBP2-PRDM16 interaction in the presence of the proteasome inhibitor MG132. By contrast, we found a substantially weaker interaction between the APPBP2-S561N mutant and PRDM16, despite comparable APPBP2 protein abundance and OGT overexpression (Fig. 2f). Consistently, reduced interaction between APPBP2-S561N and endogenous PRDM16 was observed in differentiated adipocytes ectopically expressing either APPBP2-WT or APPBP2-S561N in the presence of MG132 (Extended Data Fig.1d). It is worth noting that the reduced interaction with PRDM16 was not due to a defect in the E3 ligase complex assembly because the S561N mutation did not affect the interaction between APPBP2 and CUL2, the scaffold protein in the complex (Extended Data Fig.1e, f).

Next, we used purified proteins to determine the extent to which O-GlcNAcylation at S561 modulates APPBP2 binding affinity to PRDM16. To this end, we generated glycosylated APPBP2 protein by performing in vitro O-GlcNAcylation with recombinant OGT and purified APPBP2 proteins. As a control, we included non-glycosylated APPBP2-WT that did not undergo in vitro O-GlcNAcylation. We also generated recombinant APPBP2-S561N mutant proteins and incubated them with or without OGT. Subsequently, these APPBP2 proteins were incubated in the presence or absence of immuno-purified PRDM16 protein isolated from HEK293 cells expressing Flag-PRDM16. We found that O-GlcNAcylated APPBP2-WT displayed substantially enhanced binding to PRDM16 compared with non-modified APPBP2. On the other hand, the APPBP2-S561N mutant exhibited markedly reduced interaction with PRDM16 (Fig. 2g).

To further examine the relevance in human cells, we used CRISPR-Cas9 with homology-directed repair (HDR) to introduce the APPBP2-S561N mutation into HEK293 cells (Extended Data Fig. 1g, h). We then ectopically expressed human Flag-PRDM16 in wild-type and knock-in cells at equivalent levels (note: HEK293 cells lack endogenous PRDM16) and incubated them in either a high-glucose (25 mM) or a low-glucose (5.5 mM) medium. We found that the APPBP2-PRDM16 interaction was enhanced in wild-type cells in a high-glucose medium compared with a low-glucose medium (Fig. 2h). Notably, this glucose-dependent APPBP2-PRDM16 interaction was not observed in APPBP2^S561N^ knock-in cells. Importantly, PRDM16 protein expression was elevated in two independent APPBP2^S561N^ knock-in cells compared to that in wild-type cells (Fig. 2h; Extended Data Fig.1i).

Our previous study identified ubiquitination sites of PRDM16 through which the CUL2-APPBP2 complex ^41^. Thus, we next examined the degree to which O-GlcNAcylation of APPBP2 at S561 affects the ubiquitination of PRDM16 protein. To this end, we ectopically expressed either WT-APPBP2 or APPBP2^S561N^ together with PRDM16 and HA-tagged ubiquitin. When cells were treated with MG132 to inhibit the ubiquitin-proteasome pathway, we found that the PRDM16 protein ubiquitination was detected in cells expressing WT-APPBP2 (Fig. 2i). By contrast, much less ubiquitination signal was detected in cells expressing APPBP2^S561N^ (Fig. 2i). Together, these results suggest that high glucose induces APPBP2 O-GlcNAcylation at S561, which facilitates the association with PRDM16 and its ubiquitination by the CUL2-APPBP2 E3 ligase complex.

### O-GlcNAcylation of APPBP2 controls the PRDM16 protein stability.

To determine the role of O-GlcNAcylation of APPBP2 at S561 *in vivo*, we generated knock-in mice that carried the S561N variant in the *Appbp2* gene, herein APPBP2-KI mice (Fig. 3a). To recapitulate the human *APPBP2*^S561N^ variant, we also introduced the S562T substitution of the mouse *Appbp2* gene. Subsequently, we isolated primary stromal vascular fractions (SVF) from the inguinal WAT (iWAT) of APPBP2^S561N^ knock-in mice and littermate control mice (Fig. 3b). We found that the cultured SVFs from APPBP2^S561N^ knock-in mice differentiated to mature adipocytes at a similar degree to those from wild-type controls (Fig. 3c; Extended Data Fig. 2a). Consistent with the model, we found markedly higher PRDM16 protein levels in the inguinal WAT of APPBP2-KI mice than those from control mice despite no difference in *Prdm16* mRNA levels between the two groups (Fig. 3d; Extended Data Fig. 2a). Elevated PRDM16 protein expression was due to extended protein half-life of PRDM16 because the cycloheximide (CHX) chase experiments revealed that the half-life of the endogenous PRDM16 protein in APPBP2-KI cells (approximately 16.9 hours) was significantly longer than the control cells (approximately 7.7 hours) (Fig. 3e). These results are in agreement with our previous studies ^38, 40, 41^. Importantly, human knock-in cells carrying the APPBP2^S561N^ variant (see Fig. 2h) also showed an extended half-life of PRDM16 relative to wild-type cells (Fig. 3f). As a complementary approach, we ectopically expressed Flag-tagged OGT in differentiated adipocytes derived from SVFs from iWAT of APPBP2-KI mice and wild-type controls at equivalent levels. While OGT expression did not affect adipogenesis, OGT expression significantly reduced endogenous PRDM16 protein expression in wild-type adipocytes independent of its mRNA expression (Fig. 3g; Extended Data Fig. 2b, c). By contrast, PRDM16 protein levels were higher in APPBP2-KI adipocytes, and its levels remained unchanged even when OGT was overexpressed (Fig. 3g). Together, these results suggest that O-GlcNAcylation of APPBP2 at S561 by OGT controls the PRDM16 protein stability.

Next, we examined how APPBP2^S561N^ affects PRDM16 protein levels under two conditions where PRDM16 protein stability is reduced. The first is aging, in which the PRDM16 protein expression was significantly reduced in the iWAT, accompanied by elevated APPBP2 expression ^37, 41, 42^. At 70 weeks of age, we found that PRDM16 protein expression in the inguinal WAT of APPBP2-KI mice was higher by approximately 2-folds than that in age-matched control mice even though APPBP2 protein expression and *Prdm16* mRNA level were at similar levels between the two groups (Fig. 3h; Extended Data Fig. 2d). The second is chronic acclimation to thermoneutrality at 30°C that promotes beige-to-white fat conversion ^52, 53^. Our previous study showed that PRDM16 protein expression in the iWAT declined when mice were fed on a high-fat diet (HFD) at thermoneutrality ^41^. Following 16 weeks of acclimation to thermoneutrality on a high-fat diet, *Prdm16* mRNA expression in the iWAT remained unchanged (Extended Data Fig. 2e). However, the PRDM16 protein expression levels in APPBP2-KI mice remained higher relative to those in littermate wild-type control mice despite no difference in APPBP2 protein expression (Fig. 3i). Together, these data suggest that the APPBP2^S561N^ substitution stabilizes PRDM16 by extending its half-life *in vivo*.

### APPBP2^S561N^ knock-in mice display improved glucose homeostasis independent of body weight.

As discussed above, the Type 2 Diabetes Knowledge Portal shows that the human APPBP2^S561N^ variant (rs34146848) is associated with lower two-hour postprandial insulin and glucose levels (Fig. 4a). The development of APPBP2^S561N^ KI mice enabled us, for the first time, to determine the causal relationship. To this end, we fed APPBP2^S561N^ KI and wild-type littermate control mice at 30°C on a high-fat diet for 16 weeks. We found no difference in body weight between the genotypes, both in male and female mice (Fig. 4b, c). There was no difference in body composition, including fat mass and lean mass, between the two groups (Extended Data Fig. 3a). We also found no difference in whole-body energy expenditure, food intake, and locomotor activity between KI and control mice (Fig. 4d; Extended Data Fig. 4b-d). We note that the phenotype differed from that of adipocyte-specific APPBP2 KO mice, which exhibited higher whole-body energy expenditure and gained less body weight than control mice ^41^. The differences in energy expenditure and body weight control are likely due to the fact that APPBP2^S561N^ is a hypomorphic variant, rather than a null mutant, whereas genetic ablation of *Appbp2* potently stabilized PRDM16 protein in adipose tissue.

Nonetheless, we found that APPBP2^S561N^ KI mice showed profound protection against diet-induced glucose intolerance and insulin resistance. When male mice were maintained on a high-fat diet under thermoneutrality for 9 weeks, APPBP2^S561N^ KI mice exhibited significantly higher glucose tolerance than littermate controls (Fig. 4e). Similarly, female APPBP2^S561N^ KI mice showed improved glucose tolerance compared to female control mice at 30°C on a high-fat diet (Fig. 4f). Insulin tolerance tests showed that both male and female APPBP2^S561N^ KI mice had significantly higher insulin tolerance than littermate control mice at 30°C on a high-fat diet (Fig. 4g, h). Consistent with the observation, fasting blood glucose and serum insulin levels in both APPBP2^S561N^ KI male and female mice were lower than in control mice (Extended Data Fig. 3e, f).

Following the observation in aged mice that APPBP2^S561N^ KI mice are protective from age-associated PRDM16 proteolysis (see Fig. 3h), we next examined systemic glucose homeostasis in aged APPBP2^S561N^ KI mice. There was no difference in body weight, fat mass, or lean mass between the two groups at room temperature up to 70 weeks of age on a regular diet (Extended Data Fig. 3g, h). However, aged APPBP2^S561N^ KI mice displayed significantly improved glucose tolerance than littermate control mice (Fig. 4i). Similarly, aged APPBP2^S561N^ KI mice showed higher insulin tolerance than control mice (Fig. 4j). These results suggest that the APPBP2^S561N^ hypomorphic variant significantly affects systemic glucose homeostasis, supporting the causality between APPBP2^S561N^ and a favorable glucose profile.

### Repression of adipose tissue fibrosis and inflammation in APPBP2^S561N^ knock-in mice.

Since the APPBP2^S561N^ is expressed throughout the tissues, we sought to determine which of the metabolic organs are responsible for improved systemic glucose metabolism in APPBP2^S561N^ KI mice. To this end, we examined insulin signaling in the iWAT, interscapular BAT (iBAT), epididymal WAT (eWAT), and liver of mice that received a bolus of insulin under a fasted condition. These tissues were subjected to immunoblotting using antibodies for AKT^Ser473^ phosphorylated protein and total AKT protein. We found that the inguinal WAT of APPBP2^S561N^ KI mice showed significantly higher insulin-induced phosphorylation of AKT^Ser473^ than that of control mice (Fig. 5a). There was a trend of elevated AKT^Ser473^ phosphorylation in the iBAT of APPBP2^S561N^ KI mice, even though it did not reach statistical significance (Fig. 5b). By contrast, there was no difference in the eWAT and liver between the two groups. The data suggest that inguinal WAT is responsible for the favorable metabolic profile of APPBP2-KI mice.

We next performed RNA sequencing of the inguinal WAT to identify transcriptional changes in 70-week-old APPBP2^S561N^ KI and control mice. Biological pathway analysis of the transcriptomic data showed that APPBP2^S561N^ repressed pro-fibrosis pathways, including the extracellular matrix (ECM) organization, collagen fibril organization, wound healing, and pro-inflammatory response (Fig. 5c; Extended Data Fig. 4a-c). Under room temperature, there was no significant difference in thermogenic gene expression (Extended Data Fig. 4d). We also validated the repression of pro-fibrosis genes and thermogenic genes in APPBP2^S561N^ KI mice by qRT-PCR on independent inguinal WAT samples (Extended Data Fig. 4e, f). These results are notable because our previous study showed that the pro-fibrosis gene program, including ECM and pro-inflammatory genes, was significantly reduced in the inguinal WAT of *Appbp2* KO mice ^41^. Furthermore, the PRDM16 complex transcriptionally repress pro-fibrosis genes ^37, 39, 54^.

Excessive accumulation of ECM proteins and elevated pro-inflammatory signaling are signatures of unhealthy adipose tissue commonly observed in insulin resistance and Type 2 diabetes in humans ^5, 55-61^. Thus, we validated the observations in several independent APPBP2^S561N^ KI mouse cohorts. First, histological analyses with picrosirius red staining of adipose tissue sections showed that the inguinal WAT of 70-week-old APPBP2^S561N^ KI mice contained less collagen deposition than that of control mice (Fig. 5d, indicated by arrowheads). Second, reduced collagen deposition was observed in the inguinal WAT of APPBP2^S561N^ KI mice fed a high-fat diet for 16 weeks at 30 °C (Fig. 5e, left panel). In contrast, H&E staining of iBAT revealed no notable morphological differences between APPBP2^S561N^ KI and control mice under the same conditions (Fig. 5e, right panel). Consistent with these histological results, quantification of hydroxyproline revealed significantly lower collagen content in the inguinal WAT of male APPBP2^S561N^ KI mice than in littermate controls at 70 weeks of age (Fig. 5f). A similar reduction was observed in APPBP2^S561N^ KI mice on 16 weeks of high-fat diet at 30 ^°^C (Fig. 5g). Furthermore, this reduction was also observed in female mice on a high-fat diet at 30 C (Fig. 5h).

Next, we examined the extent to which APPBP2^S561N^ KI mice displayed altered pro-inflammatory signals in the inguinal WAT. To test this, we utilized fluorescence-activated cell sorting (FACS) to analyze the immune cell profile in WAT. The inguinal WAT from APPBP2^S561N^ KI mice showed significantly lower macrophage infiltration, based on the number of CD11b^+^ F4/80^+^ macrophages in the inguinal WAT (Fig. 5i). Furthermore, the expression of pro-inflammatory genes, including *Tgfβ*, *Nos2* and *Tnfa*, were significantly lower in the inguinal WAT of APPBP2^S561N^ KI mice compared to that of control mice (Fig. 5j). In the epidydimal WAT, there was a trend toward fewer crown-like structures and reduced macrophage infiltration in APPBP2^S561N^ KI mice than those in control mice (Extended Data Fig. 4g, h). Taken together, these data suggest that improved systemic glucose homeostasis in APPBP2^S561N^ KI mice is coupled with reduced fibrosis and inflammation and elevated insulin signaling in the inguinal WAT.

### Cell-autonomous repression of adipose tissue fibrosis in APPBP2^S561N^ knock-in mice.

To further understand the underlying mechanism, we performed Ingenuity Pathway Analysis (IPA) of the RNA-seq dataset to identify upstream regulators associated with transcriptional changes in APPBP2^S561N^ KI adipose tissues. Upstream regulator analysis predicted significant suppression of pro-inflammatory and pro-fibrotic signaling programs in APPBP2^S561N^ KI mice, including TNFα- and TGF-β pathways (Fig. 6a). Thus, we aimed to address the extent to which reduced adipose tissue fibrosis in APPBP2^S561N^ KI mice was due to reduced responses to pro-inflammatory cues in a cell-autonomous manner. To this end, we isolated primary SVFs from the inguinal WAT of APPBP2^S561N^ KI mice and control mice, which were differentiated to adipocytes in the presence or absence of TGF-(Fig. 6b). In the absence of TGF-β, both SVFs from control and KI mice differentiated into adipocytes to the same extent. However, TGF-β treatment potently inhibited adipocyte differentiation in a dose-dependent manner, whereas KI-derived cells retained a higher differentiation potency compared to the WT-derived cells (Fig. 6c). Importantly, TGF-treatment increased hydroxyproline contents in these cells in a dose-dependent fashion, but the extent of TGF-β induced hydroxyproline production was significantly lower in KI-derived adipocytes than in WT-derived cells (Fig. 6d). At the transcriptional level, TGF-β potently increased the mRNA expression of pro-fibrotic genes, including *Col1a1, Col3a1, Pcolce2,* and *Lgals3*, and in WT-derived cells, whereas the response in KI-derived adipocytes was significantly less pronounced compared to WT-derived cells (Fig. 6e). Similarly, we found that KI-derived adipocytes exhibited higher resilience to TGF-β-induced fibrotic responses than WT-derived cells when TGF-β was added in fully differentiated adipocytes for 48 hours (Extended Data Fig. 5a, b). These data are in alignment with the previous studies that PRDM16 antagonizes TGF-β-induced tissue fibrosis ^37, 39, 54^ and further suggest that the repression of adipose tissue fibrosis in APPBP2^S561N^ KI mic is mediated by reduced transcriptional responses to a pro-fibrotic cue, such as TGF-β, in a cell-autonomous manner.

Our previous study with LC-MS/MS-based proteomics of APPBP2-associated proteins in adipocytes identified PRDM16 as the most abundant protein, and the majority (87.5%) of APPBP2-linked transcription requires PRDM16 ^41^. The results indicate that PRDM16 is the dominant substrate of the CUL2-APPBP2 complex in adipocytes. Nonetheless, APPBP2 has several substrates other than PRDM16 in HEK293T cells ^46, 62^, and thus it remains unknown whether the APPBP2-mediated repressive action on pro-fibrosis genes was mediated by PRDM16. To address this, we generated inguinal WAT-derived SVFs from WT and KI mice that lacked PRDM16 using gRNAs against *Prdm16* (Fig. 6f). In line with the above result, inguinal WAT-derived SVFs from KI mice exhibited significantly lower levels of pro-fibrotic genes such as *Col1a1, Col3a1, Lgals3*, and *Pcolce2* compared to those derived from WT if PRDM16 was present. However, this difference largely disappeared in adipocytes that lacked PRDM16 (Fig. 6g). Importantly, the mRNA levels of the pro-fibrotic genes were elevated in cells lacking PRDM16. These results are in alignment with the previous study that the APPBP2-linked pro-fibrosis gene program is PRDM16-dependent.

### The metabolic improvement of APPBP2^S561N^ knock-in mice is UCP1-independent.

The best-characterized function of brown and beige adipocytes is thermogenesis by UCP1; however, emerging evidence suggests the importance of UCP1-independent pathways in systemic glucose homeostasis ^63-65^. This raises the question of the extent to which the improved systemic glucose homeostasis in APPBP2^S561N^ KI mice occurs through UCP1-independent adipose tissue remodeling. To test this, we developed APPBP2^S561N^ KI mice in the *Ucp1* KO background (APPBP2-KI x *Ucp1* KO) and littermate control mice (*Ucp1* KO) (Extended Data Fig. 6a). Consistent with the results in the wild-type background (see Fig. 4b, c), there was no difference in body weight, adiposity, and adipose tissue mass between the two groups (Fig. 7a, Extended Data Fig. 6b, c). However, we found that APPBP2^S561N^ KI × *Ucp1* KO mice exhibited markedly reduced adipose tissue fibrosis, as evidenced by decreased picrosirius red staining and lower hydroxyproline content (Fig. 7b, c). Furthermore, the transcriptional profiling found broad suppression of pro-fibrotic gene programs in APPBP2^S561N^ KI adipose tissues (Fig. 7d).

Importantly, APPBP2-KI × *Ucp1* KO mice displayed improved systemic glucose homeostasis. We found that APPBP2-KI × *Ucp1* KO mice exhibited reduced fasting blood glucose and circulating insulin levels relative to *Ucp1* KO controls on a high-fat diet (Fig. 7e, f). Furthermore, APPBP2-KI × *Ucp1* KO mice showed improved systemic glucose tolerance and insulin tolerance at room temperature (Fig. 7g, h). This metabolic phenotype is preserved when the mice were housed at 30°C (Figure 7i, j). Together, these findings demonstrate that APPBP2 S561 O-GlcNAcylation regulates systemic glucose homeostasis independently of UCP1-mediated thermogenesis, extending the importance of the UCP1-independent mechanism by which adipose tissue remodeling via the PRDM16 pathway regulates metabolic health.

## DISCUSSION

Our study identifies a post-translational mechanism linking hyperglycemia to PRDM16 protein degradation and adipose tissue dysfunction (Fig. 8). Mechanistically, glucose-dependent O-GlcNAcylation of APPBP2 at S561 enhances the interaction between APPBP2 and PRDM16, thereby facilitating PRDM16 ubiquitination and degradation by the CUL2-APPBP2 E3 ligase complex. Of note, S561 resides within an intrinsically disordered region (IDR) of APPBP2, raising the possibility that O-GlcNAcylation stabilizes the E3 complex for PRDM16 recognition and binding. Significantly, our data showed that the APPBP2^S561N^ variant, which is associated with improved metabolic traits in human genetic studies, is a hypomorphic allele with impaired O-GlcNAcylation and reduced interaction with PRDM16, leading to enhanced PRDM16 protein stability. We further demonstrated the causal relationship between the APPBP2^S561N^ variant and a favorable metabolic profile *in vivo* by developing the APPBP2^S561N^ KI mice, in which APPBP2-KI mice were protected from adipose tissue fibrosis and glucose intolerance independently of UCP1. Together, these findings suggest a model in which APPBP2 O-GlcNAcylation serves as a glucose-sensing mechanism that couples nutrient excess to PRDM16 degradation and adipose tissue remodeling toward fibrosis and inflammation.

Previous gain- and loss-of-function studies have shown that OGT promotes insulin resistance, inflammation, and tissue fibrosis. However, since OGT modifies many substrates, complete loss of OGT has pleiotropic effects that differ from those of selectively reducing APPBP2 O-GlcNAcylation. In this regard, our findings identify a molecular node through which chronic hyperglycemia promotes adipose tissue dysfunction: nutrient-sensitive O-GlcNAc signaling enhances PRDM16 degradation, leading to adipose tissue fibrosis and inflammation. Considering previous reports showing that O-GlcNAcylation modifies other E3 ligases, including NEDD4 and RAD18, it is conceivable that the glucose-sensing pathway via O-GlcNAc broadly reshapes substrate selection by ubiquitin ligases in metabolic tissues, thereby contributing to systemic metabolic dysregulation.

Another notable finding of this study is the UCP1-independent effect on adipose tissue remodeling and the impact on systemic glucose homeostasis. In mouse models, attenuation of adipose tissue fibrosis, often accompanied by beige fat biogenesis, is associated with improved systemic glucose homeostasis and insulin sensitivity ^54, 61, 66^. Consistent with these observations, our work showed that fat-specific APPBP2 deletion suppressed pro-inflammatory and fibrotic responses in the white adipose tissue of mice fed a high-fat diet ^41^. Since the magnitude of metabolic improvement by fat-selective APPBP2 deletion was disproportionately large relative to the increase in energy expenditure, we speculated that APPBP2 influences metabolic health beyond its effects on adipose tissue thermogenesis ^41^. In this study, we experimentally tested this idea by demonstrating that the metabolic improvements in APPBP2^S561N^ KI mice expressing a hypomorphic APPBP2 variant, such as reduced fibrosis and inflammation, were independent of UCP1-mediated thermogenesis. These results reinforce the concept that the metabolic improvements induced by PRDM16 protein stabilization extend beyond UCP1-mediated thermogenesis and energy expenditure, including the preservation of adipose tissue homeostasis under nutrient excess.

## METHODS

### Animal studies.

All animal experiments were approved by the Institutional Animal Care and Use Committee (IACUC; protocol #028-2022-25) at Beth Israel Deaconess Medical Center. Mice were housed under a 12-hour light–dark cycle with ad libitum access to food and water at 22 °C and ~45% humidity. UCP1 knock-out mice (Jackson Laboratory, strain #003124) and wild-type C57BL/6J mice (Jackson Laboratory, strain #000664) were used in this study. APPBP2 knock-in mice that carry the S561N mutation (equivalent to the human SNP in the APPBP2 gene) were generated by Cyagen. To recapitulate the human S561N variant of APPBP2 in mice, we mutated Ser561 to Asn and Ser562 to Thr by co-injecting the gRNA and the donor oligo containing p.Y551 (TAT to TAC) and p.A556 (GCC to GCG) into fertilized mouse oocytes. A list of the primer sequences used for genotyping and gRNA is provided in **Supplementary Table 1**.

For the sucrose drinking water model, 12-week-old male wild type C57BL/6J mouse mice were given 20% (w/v) sucrose drinking water while maintained on a standard chow diet. After 3 days of sucrose exposure, mice received daily intraperitoneal injections of Thiamet G (20 mg/kg; MedChemExpress) for an additional 3 days with continued access to sucrose water. 8 hours after the final injection, adipose tissues were harvested and processed for downstream analyses.

### Cell culture.

Stromal vascular fraction (SVF) preadipocytes were isolated from inguinal white adipose tissue (iWAT) of APPBP2 knock-in mice. To generate immortalized preadipocytes, SVF cells were infected with retrovirus expressing SV40 large T antigen as previously described ^67^. *Prdm16*-knockout preadipocytes were generated by infecting immortalized preadipocytes with eSpCas9-LentiCRISPR v2 lentivirus expressing either a scrambled control sgRNA (sgCon) or sgRNAs targeting *Prdm16*. For overexpression of OGT in APPBP2 knock-in adipocytes, immortalized preadipocytes were inflected with lentivirus carrying wild type OGT. Unless otherwise specified, adipocytes were maintained in high-glucose DMEM supplemented with 10% fetal bovine serum (FBS), 1% penicillin–streptomycin, and 1% GlutaMAX at 37 °C with 5% CO_2_. Adipocyte differentiation was induced in high-glucose DMEM supplemented with 10% FBS, 1% penicillin–streptomycin, 1% GlutaMAX, insulin (5 μg ml^−1^), triiodothyronine (T3, 1 nM), rosiglitazone (1 μM), isobutylmethylxanthine (IBMX, 0.5 mM), indomethacin (125 nM), and dexamethasone (1 μg ml^−1^). After 48 h, cells were maintained in medium containing insulin (5 μg ml^−1^), T3 (1 nM), and rosiglitazone (1 μM) for an additional 4-6 days.

### Constructs and antibody.

Cul2 (#19892), OGT-WT (#154290) and OGT-K842M (#154291) constructs were obtained from Addgene. HA-PRDM16 (human), Flag- PRDM16 (human), HA-Ub, Flag-APPBP2 (human), HA-APPBP2 (human), and APPBP2-myc (human) expression constructs were generated in-house. HA-OGT, Flag-OGT and Flag- PRDM16 (mouse) were subcloned into the pMSCV-blasticidin vector (#75085, Addgene). APPBP2 point mutants used in this study were generated by site-directed mutagenesis using the In-Fusion HD Cloning Plus kit (Takara Bio) according to the manufacturer’s instructions. All constructs were verified by Sanger sequencing.

The following antibodies were used in this study. HA antibody (3724T, Cell signaling technology), Flag antibody (2368T, Cell signaling technology), Flag antibody (14793S, Cell signaling technology), Myc antibody (2276S, Cell signaling technology), α-tubulin antibody (2125S, Cell signaling technology), β-actin antibody (A2228, Sigma-Aldrich), Vinculin antibody (sc-73614, Santa Cruze), APPBP2 antibody (NBP2-15432, Novus Biologicals), RL2 antibody (ab2739, Abcam). A custom PRDM16 antibody generated in our laboratory was used as previously described ^41^.

### Lentivirus and retrovirus production.

Retroviral and lentiviral particles were produced in Lenti-X 293T cells by co-transfection of 10 μg transfer plasmid and 10 μg packaging plasmids using SignaGen Laboratories GenJet DNA Transfection Reagent, according to the manufacturer’s instructions. Forty-eight hours after transfection, virus-containing conditioned medium was collected, filtered through a 0.45 μm membrane, and used to infect preadipocytes in the presence of 10 μg/ml polybrene for 12 h. Infected cells were subsequently selected with the indicated antibiotics (puromycin or blasticidin). For retrovirus production, gag-pol and VSV-G packaging plasmids were used; for lentivirus production, psPAX2 and pMD2.G packaging plasmids were used.

### Human knock-in cell generation.

HEK293 cells were co-transfected with the Px458 plasmid (containing SpCas9, sgRNA, and GFP) and the donor DNA template. Forty-eight hours after transfection, GFP-positive cells were sorted into single colonies with flow cytometry. Individual clones were screened, and the desired knock-in alleles were confirmed by Sanger sequencing. The sgRNA and donor DNA sequences are provided in **Supplementary Table 1**.

Homozygous SNP knock-in clones were subsequently infected with retrovirus expressing Prdm16 at equal viral titers and selected with puromycin (1 μg/ml) to generate stable Prdm16-expressing HEK293 cell lines. qRT-PCR assays conformed the same level of virus infection expressing *Prdm16*.

### Immunoprecipitation and immunoblot analyses.

Immunoprecipitation and immunoblot analyses were performed as previously described ^41^ with minor modifications. Briefly, cells were washed twice with ice-cold PBS and lysed in IP lysis buffer containing 50 mM Tris-HCl (PH 7.4), 150 mM NaCl, and 1% NP-40, supplemented with protease inhibitor cocktail. Cell lysates were incubated on ice for 15 min and clarified by centrifugation at 16,000 *g* for 15 min at 4 °C. The resulting supernatants were incubated with anti-Myc, anti-Flag or anti-HA beads (Thermo Fisher Scientific) at 4 °C for 4–6 h with gentle rotation. Beads were washed five times with IP wash buffer containing 50 mM Tris-HCl (PH 7.4), 150 mM NaCl, and 0.5% Triton X-100. Immunoprecipitated proteins were eluted by boiling the beads in SDS sample buffer and subsequently resolved by SDS–PAGE followed by immunoblot analysis using the indicated antibodies.

### O-GlcNAcylation detection.

HEK293 cells were transfected with the indicated constructs for 36 h and subsequently treated with Thiamet-G (10 μM) for 10 h. Cells were then harvested and lysed in immunoprecipitation lysis buffer (50 mM Tris-HCl, PH 7.4, 150 mM NaCl, 1% NP-40) supplemented with protease inhibitors and Thiamet-G. Cell lysates were cleared by centrifugation, and the supernatants were incubated with anti-Flag or anti-HA magnetic beads for 4 h at 4 °C. Beads were washed five times with immunoprecipitation wash buffer and subsequently boiled in SDS sample buffer. Proteins were resolved by SDS–PAGE and immunoblotted with RL2 antibody.

### Cycloheximide assay.

HEK293 cells or differentiated mature adipocytes were treated with cycloheximide (CHX) at final concentrations of 100 μg/ml or 200 μg/ml, respectively, for the indicated time periods. Cells were harvested at the indicated time points, and whole-cell lysates were prepared for immunoblot analysis of PRDM16 protein levels. β-actin or Vinculin (VCL) was used as a loading control. Band intensities were quantified using ImageJ software, and PRDM16 protein abundance was normalized to the corresponding loading control. Protein half-life was calculated based on the relative PRDM16 levels at each time point.

### Protein purification.

PRDM16 and APPBP2 proteins were purified using the SF9 insect cell expression system as previously described ^41^. SF9 cells were maintained in Sf-900 II SFM medium (Thermo Fisher Scientific) supplemented with 1% penicillin–streptomycin at 27 °C without CO_2_. His-tagged APPBP2 and Flag-tagged PRDM16 constructs were cloned into the pFastBac TOPO vector and transformed into DH10Bac competent cells to generate recombinant bacmids. Bacmids were subsequently transfected into SF9 cells using ExpiFectamine Sf Transfection Reagent to produce recombinant baculovirus, which were further amplified in Sf9 cells. For protein purification, infected SF9 cells from 1 L culture were harvested by centrifugation and stored at −80 °C. Cell pellets were lysed in buffer containing 50 mM Tris-HCl (PH 7.4), 150 mM KCl, 1 mM PMSF, 10 mM imidazole, and 0.5% Triton X-100 supplemented with protease inhibitor cocktail for 1 h at 4 °C. Lysates were homogenized, sonicated, and centrifuged at 16,000 *g* for 1 h at 4 °C. The resulting supernatants were loaded onto His GraviTrap^™^ TALON columns. The columns were washed three times with wash buffer containing 50 mM Tris-HCl (PH 7.4), 150 mM KCl, and 20 mM imidazole, and proteins were eluted in buffer containing 50 mM Tris-HCl (PH 7.4), 150 mM KCl, and 250 mM imidazole. Purified proteins were buffer-exchanged into storage buffer containing 50 mM Tris-HCl (PH 7.4), 100 mM KCl, 1 mM DTT, and 10% glycerol, aliquoted, flash-frozen, and stored at −80 °C.

### *In vitro* O-Glycosylation and binding assay.

In vitro O-GlcNAcylation assays were performed using purified APPBP2 protein and recombinant human OGT (rhOGT aa 323–1041; R&D Systems) according to the rhOGT manufacturer’s protocol with minor modifications. Briefly, purified APPBP2 protein was incubated with rhOGT in assay buffer containing 25 mM Tris-HCl (PH 7.4), 10 mM CaCl2, 10 mM MgCl2, and 0.4 mM UDP-GlcNAc (Sigma, U4375) at 37 °C for 60 min. Reactions were terminated either by addition of SDS sample buffer followed by boiling for immunoblot analysis, or buffer-exchanged into IP lysis buffer containing 50 mM Tris-HCl (PH 7.4), 150 mM NaCl, and 1% NP-40 supplemented with protease inhibitor cocktail for subsequent binding assays.

For in vitro binding assays, modified APPBP2 protein was mixed with recombinant PRDM16 protein and incubated with anti-Flag magnetic beads at 4 °C for 4–6 h with gentle rotation. Beads were washed four times with IP wash buffer containing 50 mM Tris-HCl (PH 7.4), 150 mM NaCl, and 0.5% Triton X-100. Bound proteins were eluted by boiling the beads in SDS sample buffer and analyzed by SDS–PAGE and immunoblotting.

### Glucose homeostasis analysis in mice.

Glucose homeostasis analyses were performed in aged or high-fat diet-fed male and female mice as indicated in the figure legends. For glucose tolerance tests (GTTs), mice were fasted from 9:00 to 15:00 and intraperitoneally injected with glucose at doses of 1, 1.25, or 1.5 g/kg body weight as indicated. Blood glucose levels were measured at the indicated time points before and after glucose injection. For insulin tolerance tests (ITTs), mice were fasted from 9:00 to 14:00 and intraperitoneally injected with insulin at doses of 0.75, 1, or 1.25 U kg^−1^ body weight as indicated. Blood glucose levels were measured at the indicated time points following insulin injection. Blood samples were collected from the tail vein, and glucose levels were measured using Freestyle Lite blood glucose test strips.

### Energy expenditure analysis in mice.

Whole-body energy expenditure (*V*O_2_, *V*CO_2_), food intake and locomotor activity (beam break counts) of APPBP2^S561N^ knock-in mice and littermate control mice were monitored using the Comprehensive Laboratory Animal Monitoring System (CLAMS, Columbus Instruments) with regular chow diet at 30 °C or 23 °C. Obtained indirect calorimetry data were analyzed by CaIR-ANCOVA (https://calrapp.org/), a regression-based analysis of energy expenditure in mice ^68^.

### Insulin signaling analysis *in vivo*.

APPBP2^S561N^ knock-in mice and littermate control mice were fed with high-fat diet at thermoneutral conditions (30 °C) for 8 weeks before analysis. Mice were fasted from 9:00 to 15:00 for 6 h and then intraperitoneally injected with insulin (1.5 U/kg body weight) or saline control. 10 minutes after injection, inguinal white adipose tissue (iWAT), epididymal white adipose tissue (eWAT), brown adipose tissue (BAT), and liver tissues were rapidly collected, snap-frozen in liquid nitrogen, and homogenized for protein extraction. Insulin signaling was assessed by immunoblot analysis of phosphorylated AKT (Ser473) and total AKT levels.

### Tissue histology and fibrosis measurement.

Adipose tissues were fixed in 4% paraformaldehyde overnight at 4 °C followed by dehydration in 70% ethanol. Tissues were subsequently embedded in paraffin and sectioned at 5 μm thickness. Sections were subjected to hematoxylin and eosin (H&E) staining or Picro-Sirius Red staining according to standard procedures performed by the BIDMC Pathology Core. Images were acquired using a Revolve microscope (ECHO Laboratories).

For fibrosis quantification, hydroxyproline content in adipose tissues or TGFβ-treated adipocytes was measured using a Hydroxyproline Assay Kit (Quickzyme Biosciences) according to the manufacturer’s instructions.

### Flow-cytometry analysis of adipose tissue macrophages.

Stromal vascular fractions (SVFs) were isolated from adipose tissue as previously described ^69^. Briefly, adipose tissue was dissected from mice, minced into small pieces, and digested in DMEM containing 0.1% collagenase type I supplemented with 1 mM CaCl2 at 37 °C with shaking at 180 r.p.m. for 45–60 min. Digestion was terminated by adding two volumes of DMEM, and the cell suspension was filtered through a 100-μm nylon mesh followed by centrifugation at 600g for 5 min. SVF pellets were resuspended in red blood cell lysis buffer before downstream flow cytometry analyses. SVF cells were washed with PBS and incubated with fluorochrome-conjugated antibodies diluted 1:200 in staining buffer, including PE-Cy7-conjugated anti-F4/80 (Thermo Fisher Scientific) and PE-conjugated anti-CD11b (Thermo Fisher Scientific). After staining, cells were analyzed using a Beckman Coulter CytoFLEX flow cytometer. Flow cytometry data were analyzed using FlowJo software v10.

### Lipid staining by Oil Red O.

Differentiated adipocytes were fixed in 4% paraformaldehyde for 15 min, washed once with PBS and then stained with Oil-Red-O solution (Sigma) for 10–20 min at ambient temperature. Subsequently, cells were washed three times with PBS followed by imaging with a Revolve microscope (ECHO Laboratories).

### RNA-seq and analyses

Total RNA was extracted from mouse iWAT, and purified RNA samples were subjected to commercial RNA-seq analysis (Novogene) as previously described ^69^. Four biologically independent samples per group were analyzed. Libraries were sequenced on an Illumina NovaSeq X Plus platform to generate paired-end 150-bp reads. Sequencing reads were aligned to the mouse reference genome (UCSC mm10) using STAR. Gene expression levels were quantified as fragments per kilobase of transcript per million mapped reads (FPKM). Differentially expressed genes were identified based on FPKM values, with genes showing an absolute fold change >1.5 and a *P* value <0.05 compared with controls considered differentially expressed. Functional enrichment and pathway analyses were performed using Metascape or Ingenuity Pathway Analysis (IPA).

### Gene expression analysis.

Total RNA was isolated from cells or tissues using TRIzol reagent (Invitrogen) in combination with the Direct-zol RNA Miniprep kit (Zymo Research) according to the manufacturer’s instructions. RNA was reverse-transcribed into cDNA using the iScript cDNA Synthesis Kit (Bio-Rad). Quantitative PCR was performed using a QuantStudio 6 system (Applied Biosystems). Gene expression levels were normalized to *β-actin* or *Rps18* as internal controls. Primer sequences used in this study are listed in **Supplementary Table 2**.

### Statistics and reproducibility.

All biological experiments were independently repeated at least twice with similar results. RNA-seq experiments were performed once using four biologically independent samples and validated by complementary approaches, including qRT–PCR. Statistical analyses were performed using GraphPad Prism v.10 (GraphPad Software). Data are presented as mean ± s.e.m.. Unpaired two-tailed Student’s *t*-tests were used for comparisons between two groups. One-way ANOVA followed by Tukey’s multiple-comparison test was used for comparisons among multiple groups. Two-way ANOVA was used for analyses involving two factors across multiple groups. Statistical details, including sample size and statistical tests used, are provided in the corresponding figure legends. No statistical methods were used to predetermine sample size. *P* value < 0.05 was considered statistically significant throughout the study.

## Extended Data

**Extended Data Fig. 1.**
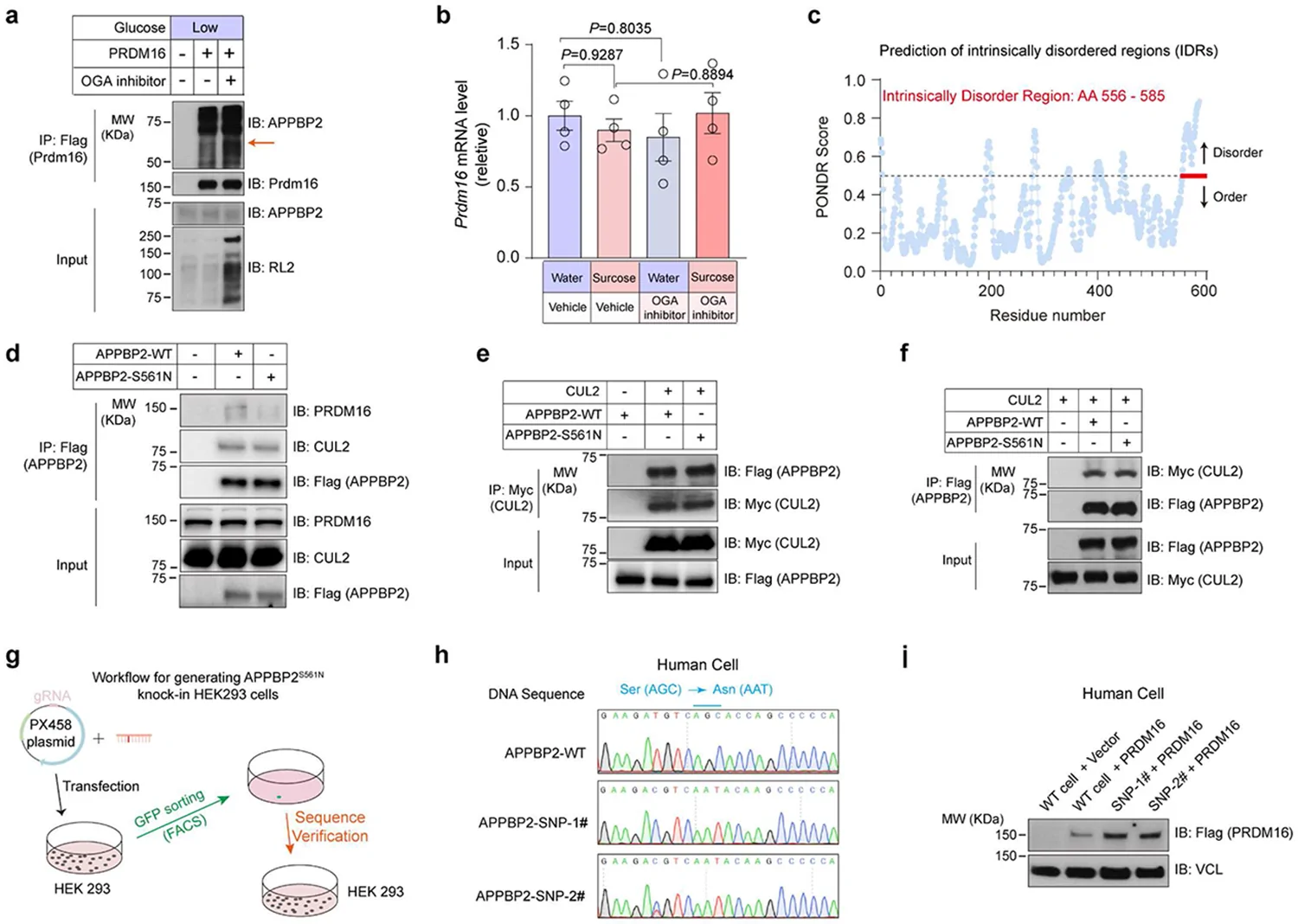
High glucose promotes PRDM16 protein degradation through the PRDM16-APPBP2 interaction. **a.** Protein interaction between ectopically expressed PRDM16 and endogenous APPBP2 in HEK293 cells treated with 5.5 mM glucose for 12 hours in the presence or absence of the OGA inhibitor Thiamet-G (10 μM). Culture medium was refreshed every 3-4 h throughout the treatment. **b.** qRT-PCR analysis of *Prdm16* mRNA levels in iWAT from sucrose-fed mice shown in Fig. 1e. *n* = 4 mice per group. Two-way ANOVA followed by Šídák’s multiple comparisons test. **c.** Predicted intrinsically disordered regions (IDRs) of human APPBP2 generated using PONDR-VSL2. **d.** Co-immunoprecipitation analysis of the interactions among APPBP2, PRDM16, and CUL2 in differentiated adipocytes ectopically expressing Flag-tagged APPBP2-WT or APPBP2-S561N. Cells were treated with MG132 (10 μM) for 12 h before harvest. Flag-tagged APPBP2 was immunoprecipitated using an anti-Flag antibody, followed by immunoblot analysis of PRDM16 and CUL2. **e.** Co-immunoprecipitation analysis of the interaction between APPBP2 and CUL2 in HEK293 cells transiently co-expressing APPBP2 and CUL2. Cells were treated with MG132 (10 μM) for 12 h before harvest. Myc-tagged CUL2 was immunoprecipitated using an anti-Myc antibody, followed by immunoblot analysis of APPBP2. **f.** Co-immunoprecipitation analysis of the interaction between APPBP2 and CUL2 in HEK293 cells transiently co-expressing APPBP2 and CUL2. Cells were treated with MG132 (10 μM) for 12 h before harvest. Flag-tagged APPBP2 was immunoprecipitated using an anti-FLAG antibody, followed by immunoblot analysis of CUL2. **g.** Schematic illustration of the generation of APPBP2-S561N knock-in (KI) HEK293 cells using CRISPR-Cas9-mediated genome editing. HEK293 cells were co-transfected with the pX458 vector expressing Cas9 and single-guide RNA (sgRNA), together with a donor DNA template carrying the S561N mutation. 48 hours after transfection, GFP-positive cells were isolated by fluorescence-activated cell sorting (FACS) and seeded as single cells for clonal expansion. Individual clones were screened, and the APPBP2-S561N mutation was confirmed by Sanger sequencing. **h.** Representative Sanger sequencing chromatograms of genomic DNA confirming the APPBP2-S561N mutation in two independent APPBP2-S561N knock-in (KI) HEK293 cell clones (APPBP2-SNP-1# and APPBP2-SNP-2#). **i.** Immunoblot analysis of PRDM16 protein levels in wild-type (WT) HEK293 cells and two independent APPBP2-S561N knock-in (KI) HEK293 cell clones (APPBP2-SNP-1# and APPBP2-SNP-2#), all stably expressing PRDM16. Vinculin (VCL) was used as a loading control.

**Extended Data Fig. 2.**
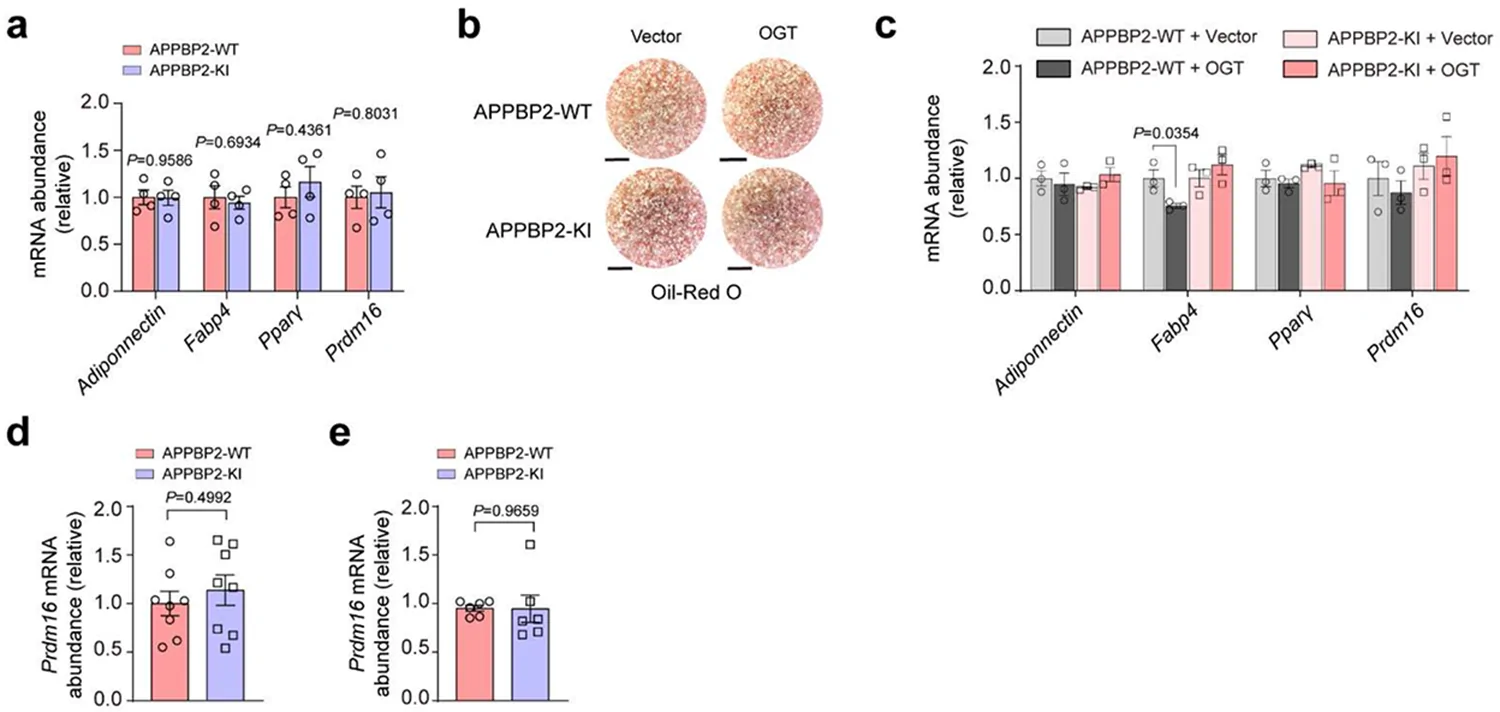
O-GlcNAcylation of APPBP2 controls the PRDM16 protein stability. **a.** qRT-PCR analysis of *Adiponectin, Fabp4, Pparγ,* and *Prdm16* mRNA levels in primary adipocytes as shown in Fig. 3b. *n* = 4 biologically independent samples per group. Data are presented as mean ± s.e.m. Unpaired two-tailed Student’s *t*-test. **b.** Representative Oil Red O staining of differentiated adipocytes stably expressing OGT or empty vector controls as shown in Fig. 3g. Scale bar, 300 μm. **c.** qRT-PCR analysis of *Adiponectin, Fabp4, Pparγ,* and *Prdm16* mRNA levels in differentiated adipocytes as shown in Fig. 3g. *n* = 3 biologically independent samples per group. Data are presented as mean ± s.e.m. Unpaired two-tailed Student’s *t*-test. **d.** qRT-PCR analysis of *Prdm16* mRNA levels in iWAT from 70-week-old APPBP2-WT and APPBP2-KI mice. *n* = 8 mice per group. Data are presented as mean ± s.e.m. Unpaired two-tailed Student’s *t*-test. **e.** qRT-PCR analysis of *Prdm16* mRNA levels in iWAT from APPBP2-WT and APPBP2-KI mice fed a high-fat diet (HFD) at 30 °C for 16 weeks. *n* = 6 mice per group. Data are presented as mean ± s.e.m. Unpaired two-tailed Student’s *t*-test.

**Extended Data Fig. 3.**
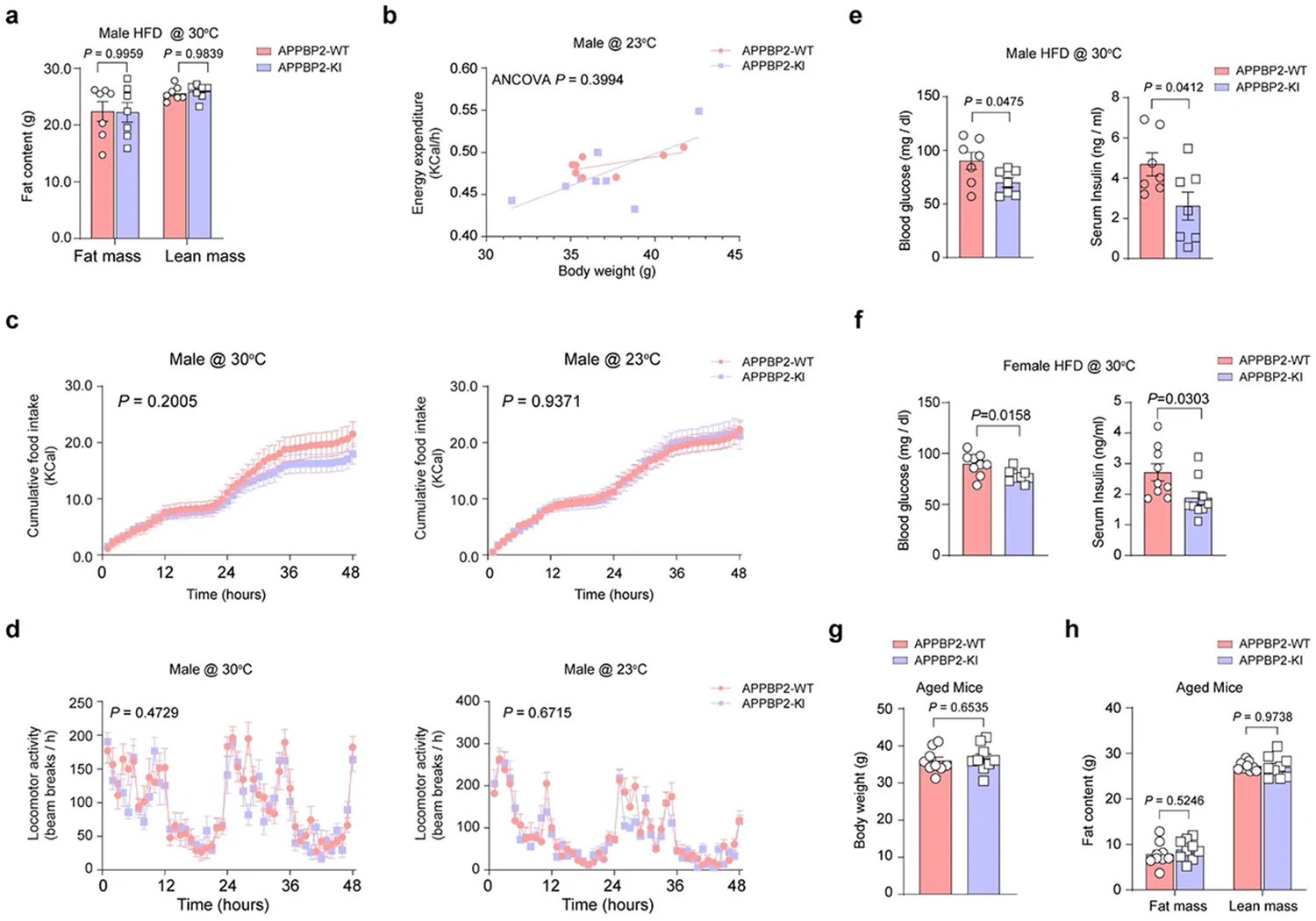
APPBP2^S561N^ knock-in mice display improved glucose homeostasis independent of body weight. **a.** Body composition analysis of fat and lean mass in male APPBP2-WT and APPBP2-KI mice fed a high-fat diet (HFD) for 16 weeks at 30 °C. *n* = 7 mice per group. Data are presented as mean ± s.e.m. Two-way ANOVA followed by Šídák’s multiple comparisons test. **b.** Energy expenditure measured by indirect calorimetry in male APPBP2-WT and APPBP2-KI mice housed at 23 °C. *n* = 8 mice per group. Statistical significance was determined by analysis of covariance (ANCOVA), with body weight included as a covariate. c. Left: Food intake of male APPBP2-WT and APPBP2-KI mice housed at 30 °C. Right: Food intake of mice housed at 23 °C. *n* = 8 mice per group. Data are presented as mean ± s.e.m. Unpaired two-tailed Student’s *t*-test. **d.** Left: Locomotor activity of male APPBP2-WT and APPBP2-KI mice housed at 30 °C. Right: Locomotor activity of mice housed at 23 °C. *n* = 8 mice per group. Data are presented as mean ± s.e.m. Unpaired two-tailed Student’s *t*-test. **e.** Overnight fasting blood glucose levels (left) and serum insulin levels (right) in male APPBP2-WT and APPBP2-KI mice after 12 weeks of HFD feeding at 30 °C. *n* = 7 mice per group. Data are presented as mean ± s.e.m. Unpaired two-tailed Student’s *t*-test. **f.** Overnight fasting blood glucose levels (left) and serum insulin levels (right) in female APPBP2-WT and APPBP2-KI mice after 14 weeks of HFD feeding at 30 °C. *n* = 9 mice per group. Data are presented as mean ± s.e.m. Unpaired two-tailed Student’s *t*-test. **g.** Body weight of 64-week-old APPBP2-WT and APPBP2-KI mice housed at 23 °C. *n* = 9 mice per group. Data are presented as mean ± s.e.m. Unpaired two-tailed Student’s *t*-test. **h.** Body composition analysis of fat mass and lean mass in 64-week-old APPBP2-WT and APPBP2-KI mice housed at 23 °C. *n* = 9 mice per group. Data are presented as mean ± s.e.m. Two-way ANOVA followed by Šídák’s multiple comparisons test.

**Extended Data Fig. 4.**
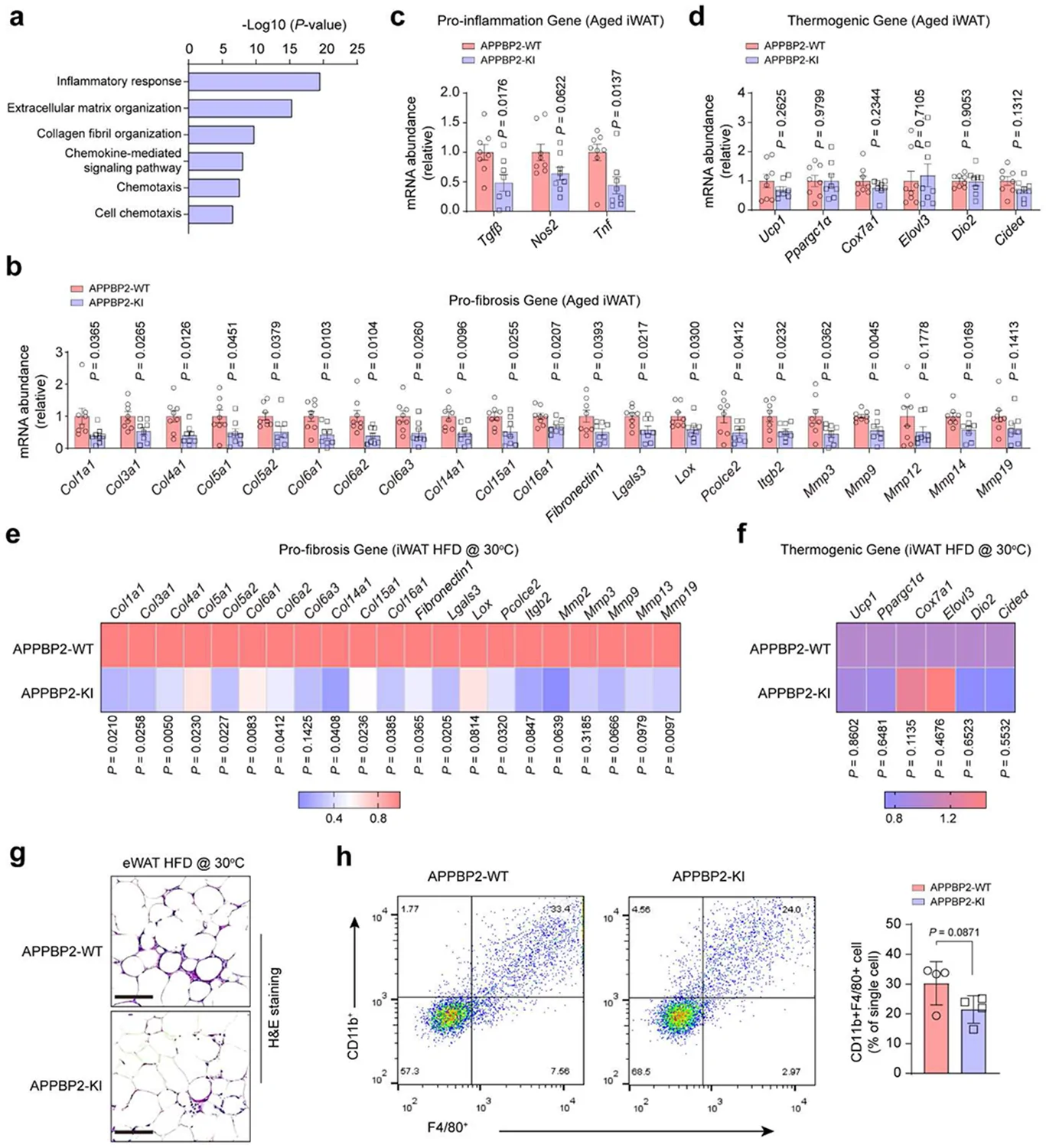
Repression of adipose tissue fibrosis and inflammation in APPBP2^S561N^ knock-in mice. **a.** GO biological process enrichment analysis showing selected biologically relevant pathways among downregulated genes in iWAT from APPBP2^S561N^ knock-in mice. **b.** qRT-PCR analysis of the indicated pro-fibrotic genes in iWAT from 70-week-old APPBP2-WT and APPBP2-KI mice. *n* = 8 mice per group. Data are presented as mean ± s.e.m. Unpaired two-tailed Student’s *t*-test. **c.** qRT-PCR analysis of *Tgfβ, Nos2*, and *Tnf* mRNA levels in iWAT from 70-week-old APPBP2-WT and APPBP2-KI mice. *n* = 8 mice per group. Data are presented as mean ± s.e.m. Unpaired two-tailed Student’s *t*-test. **d.** qRT-PCR analysis of the indicated thermogenic genes in iWAT from 70-week-old APPBP2-WT and APPBP2-KI mice. *n* = 8 mice per group. Data are presented as mean ± s.e.m. Unpaired two-tailed Student’s *t*-test. **e.** Heat map showing the indicated fibrotic gene expression profiles in iWAT from HFD-fed APPBP2-WT and APPBP2-KI mice housed at 30 °C. Data are presented as group means. Unpaired two-tailed Student’s *t*-test. **f.** Heat map showing the indicated thermogenic gene expression profiles in iWAT from HFD-fed APPBP2-WT and APPBP2-KI mice housed at 30 °C. Data are presented as group means. Unpaired two-tailed Student’s *t*-test. **g.** Representative H&E staining of eWAT from APPBP2-WT and APPBP2-KI mice fed with a high-fat diet (HFD) for 16 weeks at 30 °C. Scale bar, 210 μm. **h.** Left: Representative flow cytometry histograms of CD11b- and F4/80-expressing SVF cells isolated from eWAT of HFD-fed APPBP2-WT and APPBP2-KI mice. Right: Quantification of the percentage of CD11b^+^F4/80^+^ cells. *n* = 4 mice per group. Data are presented as mean ± s.e.m. Unpaired two-tailed Student’s *t*-test.

**Extended Data Fig. 5.**
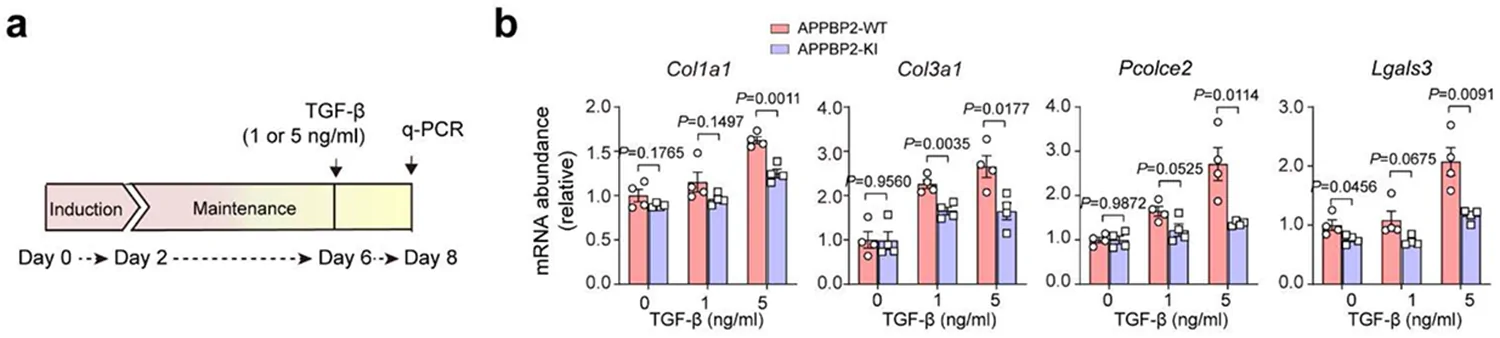
Cell-autonomous repression of adipose tissue fibrosis in APPBP2^S561N^ knock-in mice. **a.** Schematic illustration of the experimental workflow. Primary stromal vascular fraction (SVF) cells were isolated from inguinal white adipose tissue of APPBP2-WT and APPBP2-KI mice and differentiated into mature adipocytes in vitro for 6 days, followed by an additional 2 days of differentiation in the presence of the indicated concentrations of TGF-β, before qRT–PCR analysis of fibrotic genes. **b.** qRT-PCR analysis of *Col1a1, Col3a1, Pcolce2,* and *Lgals3* mRNA levels in adipocytes from (**a)**. *n* = 4 biologically independent samples per group. Data are presented as mean ± s.e.m. Unpaired two-tailed Student’s *t*-test.

**Extended Data Fig. 6.**
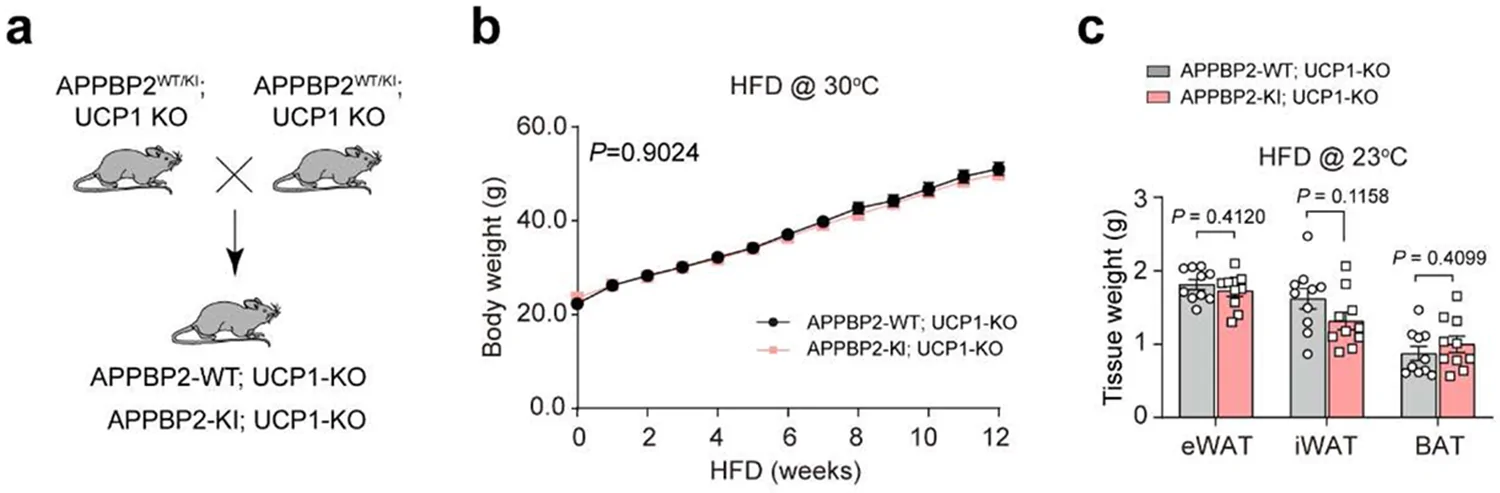
The metabolic improvement of APPBP2^S561N^ knock-in mice is UCP1-independent. **a.** Schematic illustration of the breeding strategy. APPBP2 heterozygous mice were intercrossed on a *Ucp1* knockout mouse background to generate APPBP2-KI; *Ucp1*-KO mice and littermate APPBP2-WT; *Ucp1*-KO controls. **b.** Body weight of male APPBP2-KI; *Ucp1*-KO mice and APPBP2-WT; *Ucp1*-KO control mice fed a HFD at 30 °C. *n* = 15 for control mice and 17 mice for KI mice. **c.** Tissue weights of male APPBP2-KI; *Ucp1*-KO mice and APPBP2-WT; *Ucp1*-KO control mice after 16 weeks of HFD feeding at 23 °C. *n* = 10 mice per group. Data are mean ± s.e.m. Unpaired two-tailed Student’s *t*-test.

## Supplementary Material

This is a list of supplementary files associated with this preprint. Click to download.

• SupplementaryTable.pdf

## Figures and Tables

**Fig. 1.**
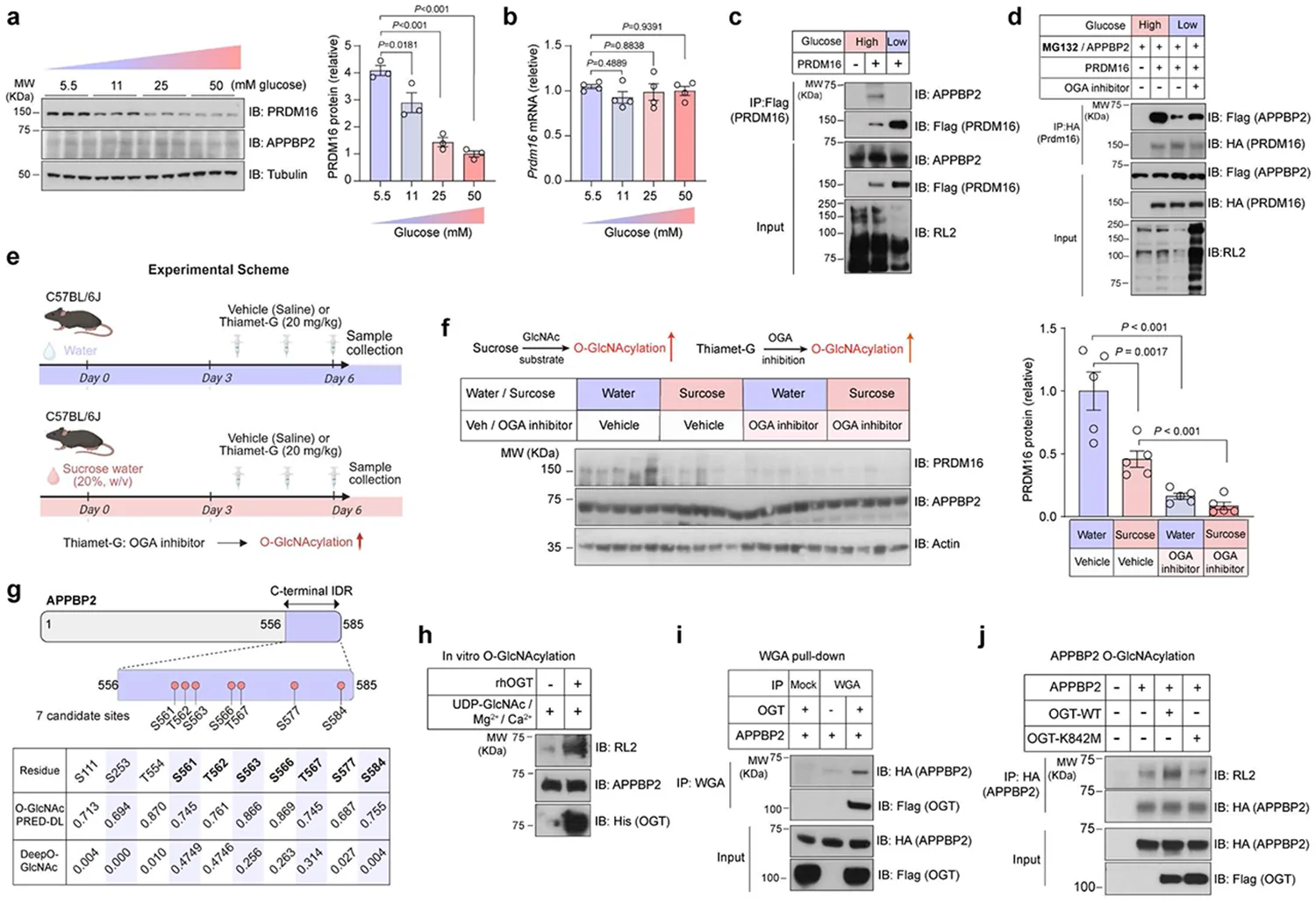
High glucose promotes PRDM16 protein degradation through enhancing the PRDM16-APPBP2 interaction. **a.** Left: Immunoblot analysis of PRDM16 and APPBP2 protein levels in HEK293 cells ectopically expressing PRDM16 after treatment with the indicated concentrations of glucose for 18 h, with culture media refreshed every 3-4 h. Right: Quantification of PRDM16 protein levels. *n* = 3 independent samples per group. Data are presented as mean ± s.e.m. One-way ANOVA followed by Tukey’s multiple comparisons test. **b.** qRT-PCR analysis of *Prdm16* mRNA levels in HEK293 cells shown in (**a**). *n* = 4 independent samples per group. Data are presented as mean ± s.e.m. One-way ANOVA followed by Tukey’s multiple comparisons test. **c.** Co-immunoprecipitation analysis of ectopically expressed PRDM16 and endogenous APPBP2 in HEK293 cells cultured with 5.5 mM (low) or 25 mM (high) glucose for 12 h. **d.** Co-immunoprecipitation analysis of ectopically expressed PRDM16 and APPBP2 in HEK293 cells cultured with 5.5 mM (low) or 25 mM (high) glucose in the presence or absence of the OGA inhibitor Thiamet-G (10 μM). **e.** Experimental scheme for OGA inhibitor (Thiamet-G) administration in sucrose-fed mice. Twelve-week-old male C57BL/6J mice were maintained on chow diet at room temperature and provided with 20% sucrose drinking water for 3 days, followed by daily intraperitoneal injection of Thiamet-G (20 mg/kg) for an additional 3 days. Inguinal white adipose tissue (iWAT) was collected 8 hours after the final injection. **f.** Left: Immunoblot analysis of PRDM16 and APPBP2 protein levels in iWAT from **(e)**. Actin was used as a loading control. Right: Quantification of PRDM16 protein levels. *n* = 5 mice per group. Data are presented as mean ± s.e.m. Two-way ANOVA followed by Šídák’s multiple comparisons test. **g.** Upper: Schematic representation of human APPBP2 showing seven candidate O-GlcNAcylation sites within the C-terminal intrinsically disordered region (residues 556–585; Extended Data Fig. 1c). Lower: Predicted O-GlcNAcylation scores from O-GlcNAcPRED-DL (accessed November 2024) and DeepO-GlcNAc. Sites within the IDR that were predicted as positive by O-GlcNAcPRED-DL were selected. **h.** *In vitro* reconstitution of APPBP2 O-GlcNAcylation using recombinant human O-GlcNAc transferase (rhOGT) and APPBP2 protein in the presence of Mg^2+^, Ca^2+^ and UDP-GlcNAc substrate. APPBP2 O-GlcNAcylation was assessed by immunoblot analysis using the O-GlcNAc-specific antibody RL2. **i.** Analysis of APPBP2 O-GlcNAcylation by wheat germ agglutinin (WGA) pull-down assay in HEK293 cells expressing APPBP2 in the presence or absence of O-GlcNAc transferase (OGT). **j.** Analysis of APPBP2 O-GlcNAcylation in HEK293 cells transiently expressing APPBP2 together with wild-type OGT (OGT-WT) or its catalytically inactive mutant (OGT-K842M). APPBP2 was immunoprecipitated, and its O-GlcNAcylation was assessed by immunoblot analysis using the O-GlcNAc-specific antibody RL2.

**Fig. 2.**
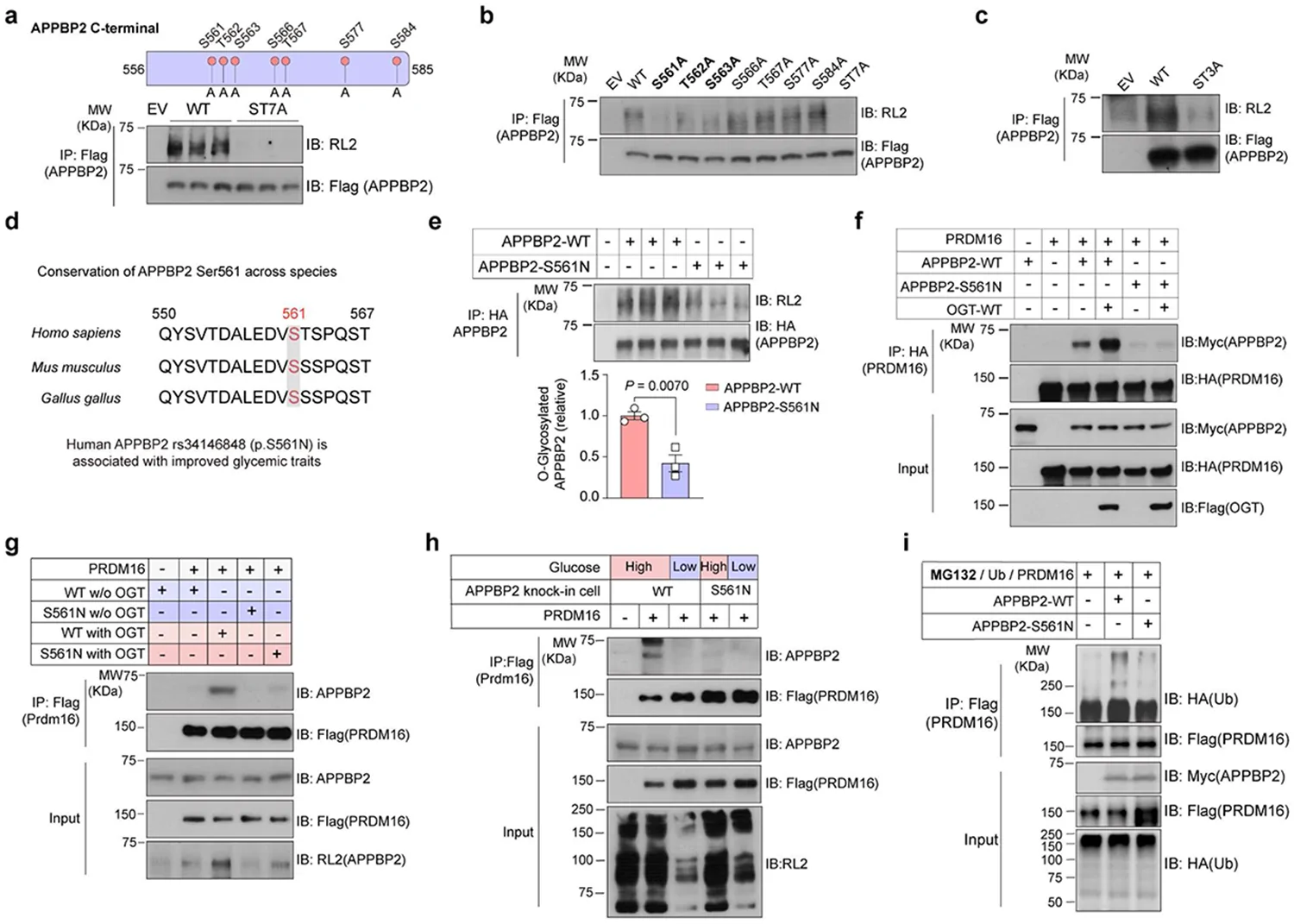
Identification of glucose-sensitive O-GlcNAcylation of APPBP2. **a.** Upper: Schematic representation of human APPBP2 showing seven candidate O-GlcNAcylation sites. Lower: Analysis of O-GlcNAcylation of wild-type APPBP2 (APPBP2-WT) and the seven-site alanine-substitution mutant (APPBP2-ST7A; S561A/T562A/S563A/S566A/T567A/S577A/S584A) in HEK293 cells stably expressing wild-type O-GlcNAc transferase (OGT-WT). APPBP2 was immunoprecipitated, and its O-GlcNAcylation was assessed by immunoblot analysis using the O-GlcNAc-specific antibody RL2. **b.** Analysis of O-GlcNAcylation of the indicated APPBP2 single-site mutants and the seven-site mutant (ST7A) in HEK293 cells stably expressing OGT-WT. **c.** Analysis of O-GlcNAcylation of APPBP2-WT and the triple-site alanine-substitution mutant (APPBP2-ST3A; S561A/T562A/S563A) in HEK293 cells stably expressing OGT-WT. **d.** Sequence alignment of APPBP2 amino acid residues Q550–T567 from human, mouse and chicken, highlighting the evolutionary conservation of S561. The human APPBP2 genetic variant rs34146848 (p.S561N), associated with improved glycemic traits, is indicated. **e.** Upper: Analysis of APPBP2-WT and APPBP2-S561N O-GlcNAcylation in HEK293 cells stably expressing OGT-WT. Lower: Quantification of O-GlcNAcylated APPBP2 levels. *n* = 3 independent samples per group. Data are presented as mean ± s.e.m. Unpaired two-tailed Studenťs *t*-test. **f.** Co-immunoprecipitation analysis of the interaction between ectopically expressed PRDM16 and APPBP2 in HEK293 cells stably expressing OGT-WT. Cells were treated with MG132 (10 μM) for 12 h before harvest. **g.** *In vitro* co-immunoprecipitation analysis of the interaction between recombinant PRDM16 and unmodified or O-GlcNAcylated APPBP2. Purified recombinant APPBP2 was incubated with recombinant human OGT under O-GlcNAcylation reaction conditions as described in Fig. 1h. Following the reaction, samples were dialyzed against immunoprecipitation (IP) buffer and subsequently incubated with purified recombinant PRDM16. The interaction between PRDM16 and APPBP2 was assessed by immunoprecipitation followed by immunoblot analysis. **h.** Co-immunoprecipitation analysis of the interaction between ectopically expressed PRDM16 and endogenous APPBP2 in wild-type (WT) or APPBP2-S561N knock-in (KI) HEK293 cells stably expressing PRDM16. Cells were cultured with low (5.5 mM) or high (25 mM) glucose for 12 h before harvest. **i.** Analysis of PRDM16 polyubiquitination mediated by APPBP2-WT or APPBP2-S561N. HEK293 cells were transiently transfected with PRDM16, ubiquitin (Ub), and either APPBP2-WT or APPBP2-S561N. PRDM16 polyubiquitination was assessed by immunoprecipitation of PRDM16 followed by immunoblot analysis. Cells were treated with MG132 (10 μM) for 12 h before harvest.

**Fig. 3.**
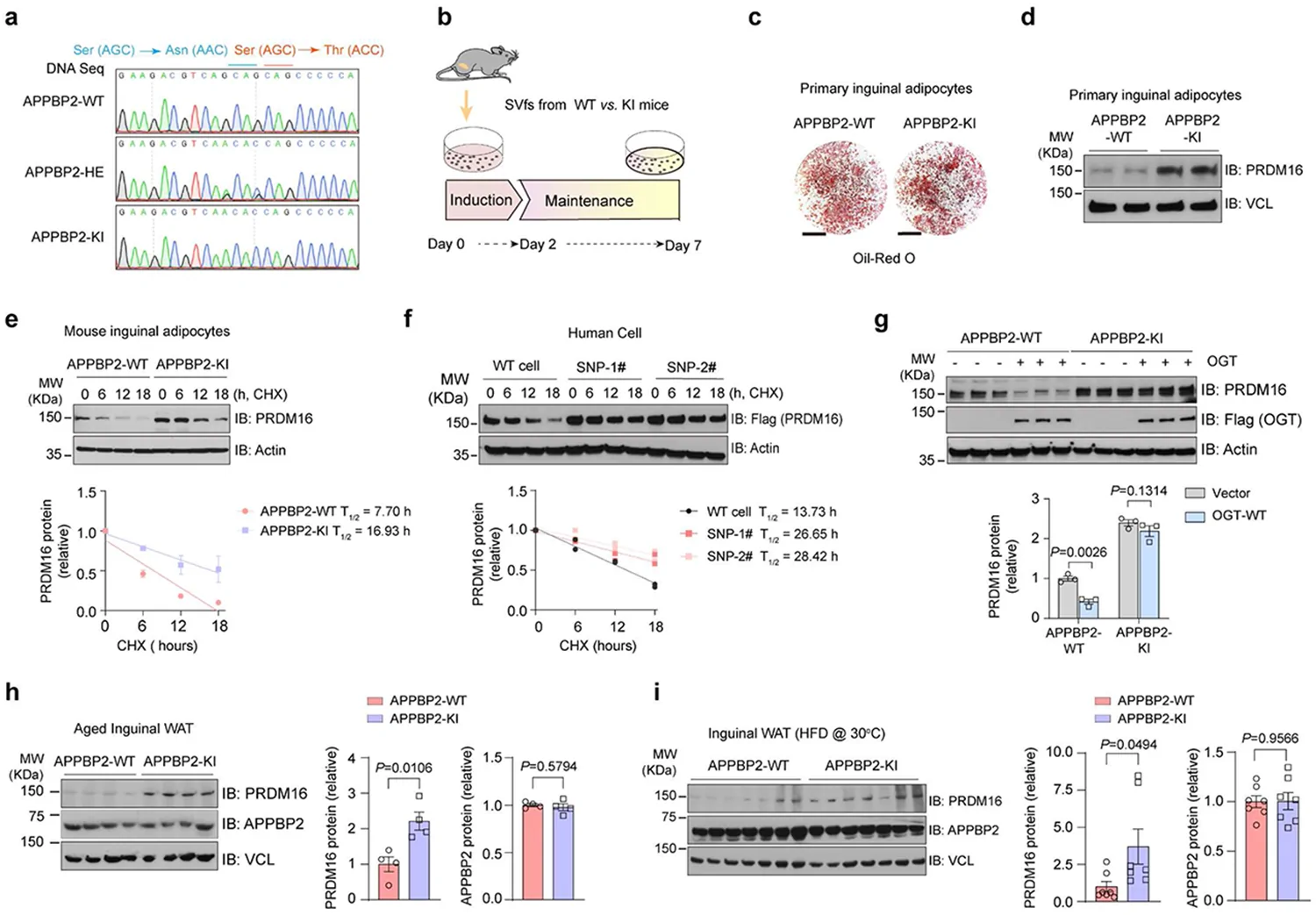
O-GlcNAcylation of APPBP2 controls the PRDM16 protein stability. **a.** Sanger sequencing confirms APPBP2^S561N^ knock-in (APPBP2-KI) mice using genomic DNA. Note that the C-terminal amino acid sequence of APPBP2 is highly conserved between mouse and human, except for residue T562 (corresponding to S562 in mouse APPBP2; see Fig. 2d). To recapitulate the human APPBP2-S561N variant in mice, S561N and S562T mutations were introduced into mouse APPBP2. **b.** Schematic illustration of the experimental workflow. Primary stromal vascular fraction (SVF) cells were isolated from inguinal white adipose tissue (iWAT) of APPBP2-WT and APPBP2-KI mice, differentiated into mature adipocytes for 7 days in vitro, and subjected to the downstream analyses. **c.** Representative Oil Red O staining of differentiated primary adipocytes as shown in (**b**). Scale bar, 300 μm. **d.** Immunoblot analysis of PRDM16 protein levels in differentiated adipocytes from (**b**). VCL (Vinculin) was used as a loading control. **e.** Upper: Immunoblot analysis of PRDM16 protein stability following the indicated cycloheximide (CHX) treatment in differentiated adipocytes from (**b**). Lower: Quantification of endogenous PRDM16 protein stability in APPBP2-WT and APPBP2-KI primary inguinal adipocytes. *n* = 2 independent experiments. Data are presented as mean ± s.e.m. **f.** Upper: Immunoblot analysis of PRDM16 protein stability following the indicated CHX treatment in APPBP2-S561N knock-in or APPBP2-WT HEK293 cells stably expressing PRDM16. Actin was used as a loading control. Lower: Quantification of PRDM16 protein stability in indicated HEK293 cells. *n* = 2 independent experiments. Data are presented as mean ± s.e.m. **g.** Upper: Immunoblot analysis of PRDM16 in immortalized inguinal white adipocytes stably overexpressing OGT. Actin was used as a loading control. Lower: Quantification of PRDM16 protein levels. *n* = 3 biologically independent samples. Data are presented as mean ± s.e.m. Two-way ANOVA followed by Šídák’s multiple comparisons test. **h.** Left: Immunoblot analysis of PRDM16 and APPBP2 protein levels in iWAT from 70-week-old APPBP2-WT and APPBP2-KI mice. Right: Quantification of PRDM16 and APPBP2 protein levels. *n* = 4 mice per group. Data are presented as mean ± s.e.m. Unpaired two-tailed Student’s *t-*test. **i.** Left: Immunoblot analysis of PRDM16 and APPBP2 protein levels in iWAT from high-fat diet (HFD)-fed APPBP2-WT and APPBP2-KI mice housed at 30 °C. Right: Quantification of PRDM16 and APPBP2 protein levels. *n* = 7 mice per group. Data are presented as mean ± s.e.m. Unpaired two-tailed Student’s *t*-test.

**Fig. 4.**
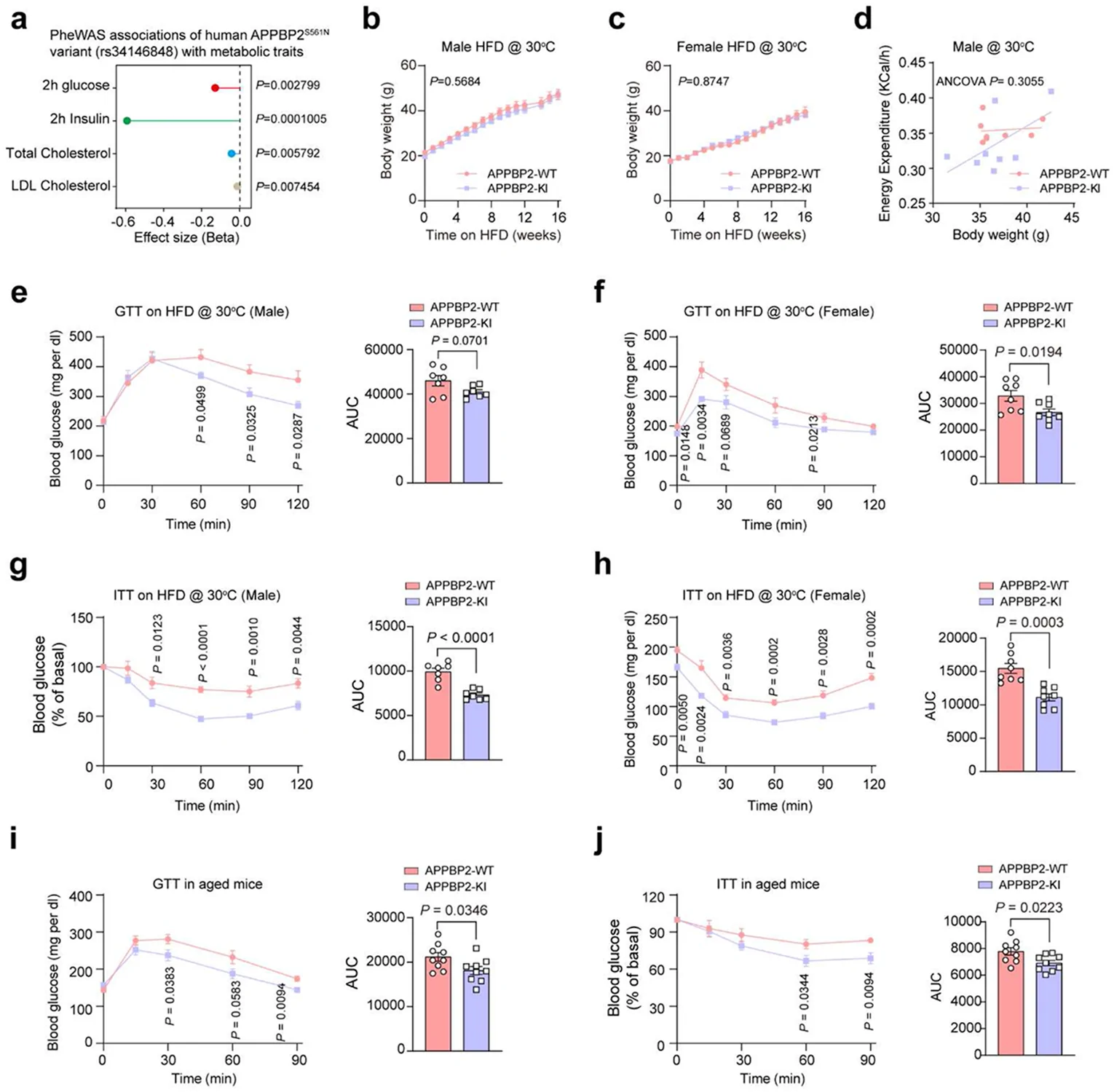
APPBP2^S561N^ knock-in mice display improved glucose homeostasis independent of body weight. **a.** PheWAS associations of the human APPBP2 S561N variant (rs34146848) with metabolic traits from the Human T2D Knowledge Portal. Dots indicate effect size (beta), and horizontal lines represent 95% confidence intervals. *P* values are indicated on the right. **b.** Body weight of male APPBP2-KI mice and APPBP2-WT (controls) on a high fat diet (HFD) at 30 °C. *n* = 7 mice per group. Data are presented as mean ± s.e.m. Unpaired two-tailed Student’s *t-*test. **c.** Body weight of female APPBP2-KI mice and APPBP2-WT (controls) on a HFD at 30 °C. *n* = 9 mice per group. Data are presented as mean ± s.e.m. Unpaired two-tailed Student’s *t*-test. **d.** Regression-based analysis of energy expenditure against body mass between APPBP2-WT and APPBP2-KI mice at 30°C. *n* = 8 mice per group. Data were analyzed using CaIR-ANCOVA with energy expenditure as a dependent variable and body mass as a covariate. *P* values are shown at the top. **e.** Left: Glucose tolerance test (GTT) in male mice after 9 weeks of HFD feeding at 30 °C. *n* = 7 mice per group. Right: Quantification of GTT by area under the curve (AUC). Unpaired two-tailed Student’s *t*-test (GTT, at individual time points without multiple-comparison correction; AUC, between groups). **f.** Left: Glucose tolerance test in female mice after 11 weeks of HFD at 30 °C. *n* = 8 mice per group. Right: Quantification of GTT by AUC. Unpaired two-tailed Student’s *t*-test (GTT, at individual time points without multiple-comparison correction; AUC, between groups). **g.** Left: Insulin tolerance test (ITT) in male mice after 10 weeks of HFD at 30 °C. *n* = 7 mice per group. Right: Quantification of ITT by AUC. Unpaired two-tailed Student’s *t*-test (ITT, at individual time points without multiple-comparison correction; AUC, between groups). **h.** Left: Insulin tolerance test in female mice after 12 weeks of HFD at 30 °C. *n* = 8 mice per group. Right: Quantification of ITT by AUC. Unpaired two-tailed Student’s *t*-test (ITT, at individual time points without multiple-comparison correction; AUC, between groups). **i.** Left: Glucose tolerance test in 64 weeks old aged male mice. *n* = 9 mice per group. Right: Quantification of GTT by AUC. Unpaired two-tailed Student’s *t*-test (GTT, at individual time points without multiple-comparison correction; AUC, between groups). **j.** Left: Insulin tolerance test in 64 weeks old aged male mice in (**i**). Right: Quantification of ITT by AUC. Unpaired two-tailed Student’s *t*-test (ITT, at individual time points without multiple-comparison correction; AUC, between groups).

**Fig. 5.**
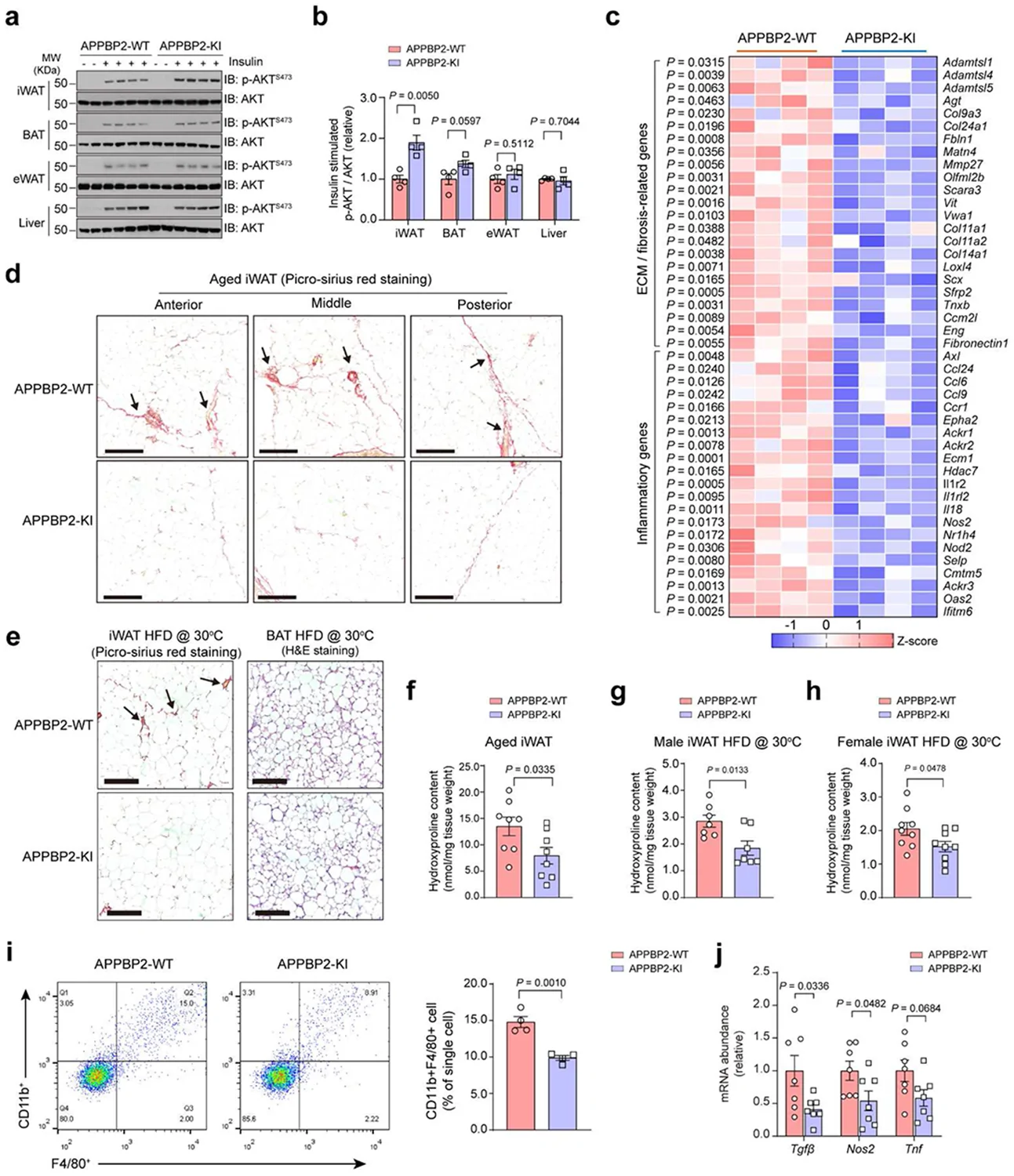
Repression of adipose tissue fibrosis and inflammation in APPBP2^S561N^ knock-in mice. **a.** Insulin signaling in metabolic organs of APPBP2^S561N^ knock-in mice. Mice were fed a high-fat diet (HFD) at 30 °C for 10 weeks, followed by intraperitoneal injection of insulin (1.5 U/kg body weight) for 10 min before rapid tissue collection. Tissue lysates from the indicated organs were subjected to immunoblotting analysis of p-AKT^S473^ and total AKT. *n* = 2 mice per genotype in the control group and *n* = 4 mice per genotype in the insulin-treated group. **b.** Quantification of insulin stimulated p-AKT^S473^/AKT ratio shown in (**a**). Data are presented as mean ± s.e.m. Unpaired two-tailed Student’s *t*-test. **c.** Heat map showing reduced extracellular matrix (ECM), pro-fibrotic, and pro-inflammatory gene profiles in the inguinal white adipose tissue (iWAT) of aged APPBP2^S561N^ knock-in mice. *n* = 4 mice per group. *P* values shown on the left were calculated by unpaired two-tailed Student’s *t*-test. **d.** Representative Picro-Sirius Red staining of iWAT from aged APPBP2^S561N^ knock-in mice. Arrowheads indicate areas of collagen deposition. Scale bar, 210 μm. **e.** Left: Representative Picro-Sirius Red staining of iWAT from high-fat diet (HFD)-fed APPBP2^S561N^ knock-in mice at 30 °C. Arrowheads indicate areas of collagen deposition. Right: Representative hematoxylin and eosin (H&E) staining of brown adipose tissue from high-fat diet (HFD)-fed APPBP2^S561N^ knock-in mice at 30 °C. Scale bar, 210 μm. **f.** Hydroxyproline content in iWAT from aged male APPBP2^S561N^ knock-in mice. *n* = 8 mice per group. Data are presented as mean ± s.e.m. Unpaired two-tailed Student’s *t*-test. **g.** Hydroxyproline content in iWAT from male APPBP2^S561N^ knock-in mice fed a HFD at 30 °C for 16 weeks. *n* = 7 mice per group. Data are presented as mean ± s.e.m. Unpaired two-tailed Student’s *t*-test. **h.** Hydroxyproline content in iWAT from female APPBP2^S561N^ knock-in mice fed a HFD at 30 °C for 16 weeks. *n* = 9 mice per group. Data are presented as mean ± s.e.m. Unpaired two-tailed Student’s *t*-test. **i.** Left: Representative flow cytometry histograms of CD11b- and F4/80-expressing SVF cells isolated from iWAT of HFD-fed mice. Right: Quantification of the percentage of CD11b^+^ and F4/80^+^ cells. *n* = 4 mice per group. Data are presented as mean ± s.e.m. Unpaired two-tailed Student’s *t*-test. **j.** qRT-PCR analysis of the indicated inflammatory genes in iWAT from male APPBP2^S561N^ knock-in mice fed a HFD at 30 °C. *n* = 7 mice per group. Data are presented as mean ± s.e.m. Unpaired two-tailed Student’s *t*-test.

**Fig. 6.**
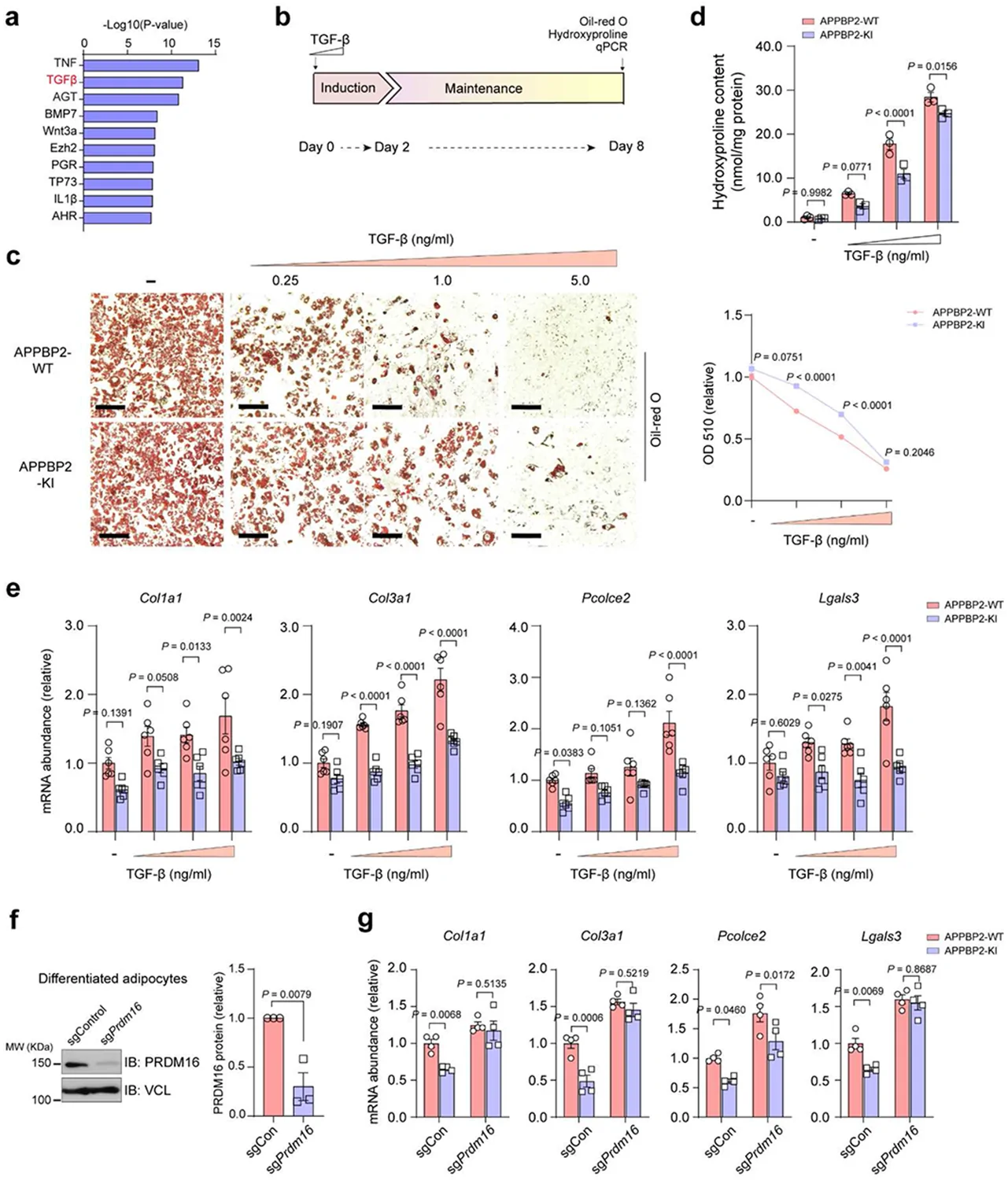
Cell-autonomous repression of adipose tissue fibrosis in APPBP2^S561N^ knock-in mice. **a.** Ingenuity upstream analysis identified repressed signaling pathways in the iWAT of aged APPBP2^S561N^ knock-in mice. **b.** Schematic illustration of the experimental workflow. Primary stromal vascular fraction (SVF) cells were isolated from inguinal white adipose tissue (iWAT), differentiated into mature adipocytes in vitro for 8 days in the presence of the indicated concentrations of TGF-β during differentiation, and subjected to the indicated downstream analyses. **c.** Left: Representative Oil Red O staining of cells from (b) after 8 days of differentiation in the presence of TGF-β treatment. Right: Quantification of Oil Red O staining as indicated by OD510. *n* = 3 biologically independent samples per group. Data are presented as mean ± s.e.m. Two-way ANOVA followed with Šídák's multiple comparisons test. **d.** Hydroxyproline content of cells from (**b**) after 8 days of differentiation in the presence of TGF-β treatment. *n* = 3 biologically independent samples per group. Data are presented as mean ± s.e.m. Two-way ANOVA followed with Šídák's multiple comparisons test. **e.** qRT-PCR analysis of *Col1a1, Col3a1, Pcolce2*, and *Lgals3* mRNA levels in cells from (**b**) after 8 days of differentiation in the presence of TGF-β treatment. *n* = 6 biologically independent samples per group. Data are presented as mean ± s.e.m. Two-way ANOVA followed with Šídák's multiple comparisons test. **f.** Left: Immunoblot analysis of PRDM16 protein levels in differentiated adipocytes stably expressing sgCon or sg*Prdm16*. Right: Quantification of PRDM16 protein levels. *n* = 3 independent experiments. Data are presented as mean ± s.e.m. Unpaired two-tailed Student’s *t*-test. **g.** qRT-PCR analysis of *Col1a1, Col3a1, Pcolce2*, and *Lgals3* mRNA levels in differentiated sgCon and sg*Prdm16* adipocytes treated with TGF-β (5 ng/ml) for 2 days. *n* = 4 biologically independent samples per group. Data are presented as mean ± s.e.m. Two-way ANOVA followed by Šídák's multiple comparisons test.

**Fig. 7.**
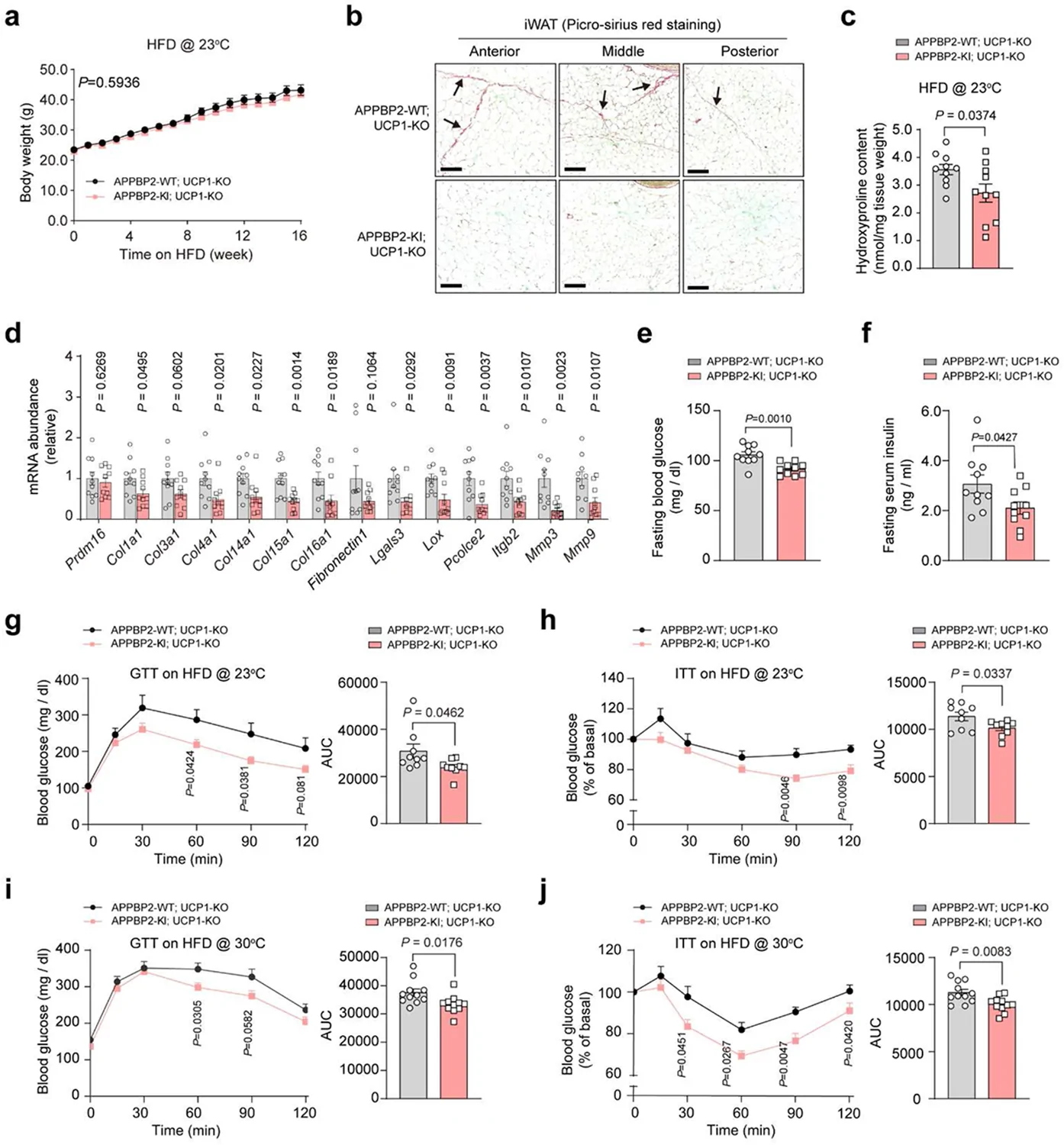
The metabolic improvement of APPBP2^S561N^ knock-in mice is UCP1-independent. **a.** Body weight of male APPBP2-KI; *Ucp1*-KO mice and APPBP2-WT; *Ucp1*-KO control mice fed a high-fat diet (HFD) at room temperature (23 °C). *n* = 10 mice per group. Data are presented as mean ± s.e.m. Unpaired two-tailed Student’s *t*-test. **b.** Representative Picro-sirius red staining of inguinal white adipose tissue (iWAT) from mice fed an HFD for 16 weeks. Arrowheads indicate areas of collagen deposition. Scale bar, 210 μm. **c.** Hydroxyproline content of iWAT from male mice fed an HFD for 16 weeks. *n* = 10 mice per group. Data are presented as mean ± s.e.m. Unpaired two-tailed Student’s *t*-test. **d.** qRT-PCR analysis of *Prdm16* and indicated pro-fibrotic genes in iWAT from mice fed an HFD for 16 weeks. *n* = 10 mice per group. Data are presented as mean ± s.e.m. Unpaired two-tailed Student’s *t*-test. **e.** Overnight fasting blood glucose levels after 12 weeks of HFD feeding. *n* = 10 mice per group. Data are presented as mean ± s.e.m. Unpaired two-tailed Student’s *t*-test. **f.** Overnight fasting serum insulin levels after 12 weeks of HFD feeding. *n* = 10 mice per group. Data are presented as mean ± s.e.m. Unpaired two-tailed Student’s *t*-test. **g.** Left: Glucose tolerance test (GTT) in male mice after 10 weeks of HFD feeding at 23 °C. *n* = 9 mice per group. Right: Quantification of GTT by area under the curve (AUC). Data are presented as mean ± s.e.m. Unpaired two-tailed Student’s *t*-test (GTT, at individual time points without multiple-comparison correction; AUC, between groups). **h.** Left: Insulin tolerance test (ITT) in male mice after 11 weeks of HFD feeding at 23 °C. *n* = 9 mice per group. Right: Quantification of ITT by area under the curve (AUC). Data are presented as mean ± s.e.m. Unpaired two-tailed Student’s *t*-test (ITT, at individual time points without multiple-comparison correction; AUC, between groups). **i.** Left: Glucose tolerance test (GTT) in male mice after 12 weeks of HFD feeding at 30 °C. *n* = 11 mice per group. Right: Quantification of GTT by area under the curve (AUC). Data are presented as mean ± s.e.m. Unpaired two-tailed Student’s *t*-test (GTT, at individual time points without multiple-comparison correction; AUC, between groups). **j.** Left: Insulin tolerance test (ITT) in male mice after 13 weeks of HFD feeding at 30 °C. *n* = 11 mice per group. Right: Quantification of ITT by area under the curve (AUC). Data are presented as mean ± s.e.m. Unpaired two-tailed Student’s *t*-test (ITT, at individual time points without multiple-comparison correction; AUC, between groups).

**Fig.8.**
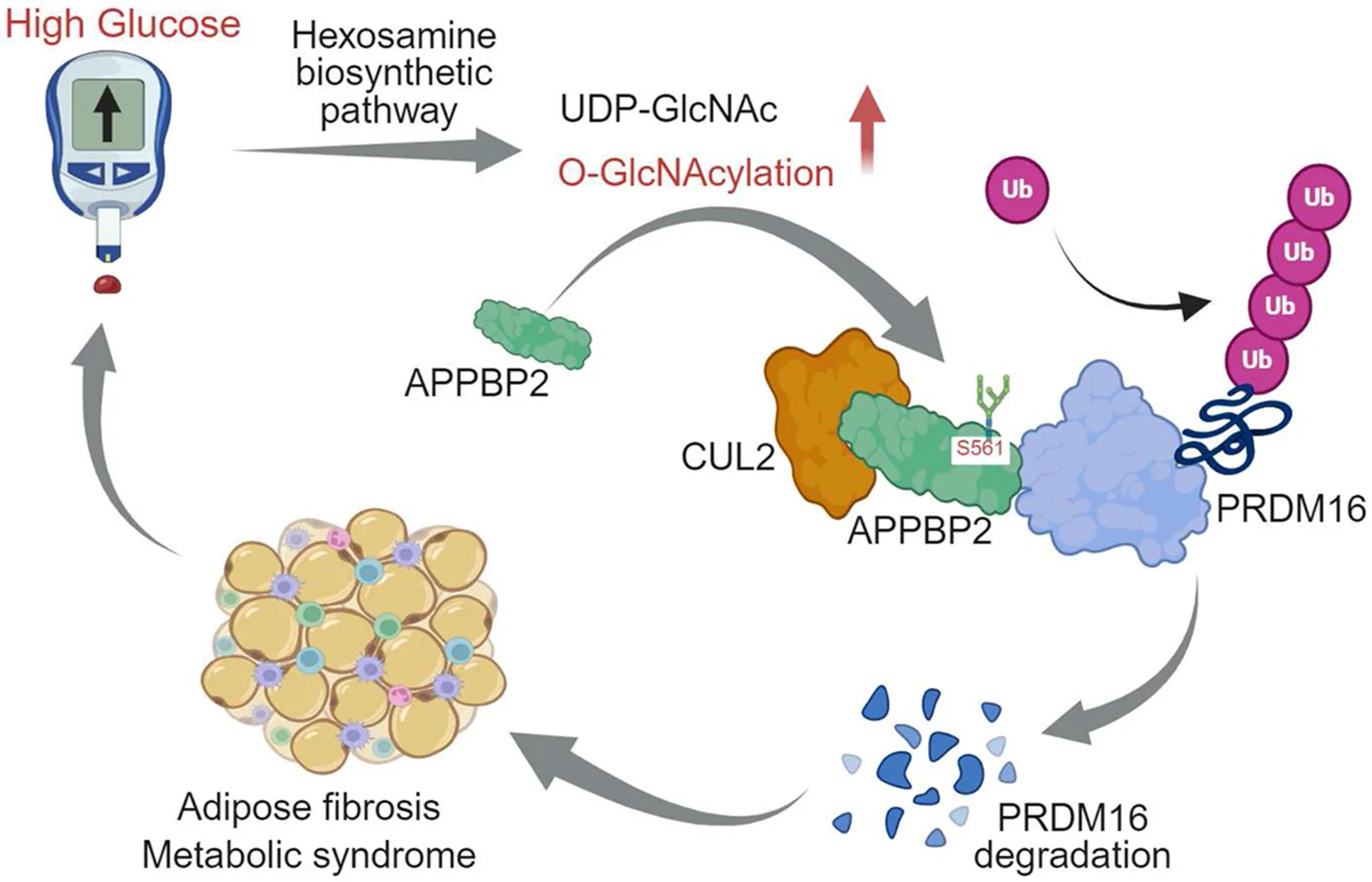
A proposed model by which hyperglycemia promotes adipose tissue dysfunction. APPBP2 O-GlcNAcylation at Ser561 through the hexosamine biosynthetic pathway (HBP) promotes its interaction with PRDM16, which facilitates PRDM16 polyubiquitination and proteasomal degradation through the E3 ubiquitin ligase, the CUL2-APPBP2 complex. Enhanced PRDM16 degradation contributes to adipose tissue dysfunction, characterized by increased fibrosis and inflammation, ultimately impairing systemic metabolic homeostasis. Notably, the APPBP2 S561N variant (rs34146848), which is associated with improved glucose homeostasis in humans, is a hypo-morphic variant that is resistant to high glucose-induced O-GlcNAcylation, thereby leading to enhanced PRDM16 stability. In agreement, APPBP2 ^S561N^ knock-in mice exhibited improved glucose homeostasis and protection against adipose tissue fibrosis and inflammation.

## Data Availability

RNA-seq data have been deposited in the Gene Expression Omnibus under accession number GSE331400. All other data are available in the manuscript or supplementary materials. All the materials and mice are available after completion of MTA.
